# Identification of potential therapeutic targets for stroke and its subtypes by integrating proteomes and genetics from human plasma

**DOI:** 10.1093/braincomms/fcaf457

**Published:** 2025-12-30

**Authors:** Hanchen Liu, Xiaoxi Zhang, Hongyu Ma, Thanh N Nguyen, Yi Ling, Shaojun Mo, Qinghai Huang, Jianmin Liu, Yu Zhou, Pengfei Yang

**Affiliations:** Department of Neurosurgery, Beijing Tiantan Hospital, Capital Medical University, Beijing 100070, China; Neurovascular Center, Naval Medical University Changhai Hospital, Shanghai 200433, China; Neurovascular Center, Naval Medical University Changhai Hospital, Shanghai 200433, China; Neurovascular Center, Naval Medical University Changhai Hospital, Shanghai 200433, China; Boston University Chobanian and Avedisian School of Medicine, Department of Neurology & Radiology, Boston Med Center, Boston, MA 02118, USA; Neurovascular Center, Naval Medical University Changhai Hospital, Shanghai 200433, China; Regiment 2, College of Basic Medicine, Naval Medical University, Shanghai 200433, China; Neurovascular Center, Naval Medical University Changhai Hospital, Shanghai 200433, China; Neurovascular Center, Naval Medical University Changhai Hospital, Shanghai 200433, China; Neurovascular Center, Naval Medical University Changhai Hospital, Shanghai 200433, China; Neurovascular Center, Naval Medical University Changhai Hospital, Shanghai 200433, China; Neurovascular Center, Naval Medical University Changhai Hospital, Shanghai 200433, China

**Keywords:** stroke, proteome-wide association study, transcriptome-wide association study, Bayesian colocalization, human plasma proteomes

## Abstract

Previous genome-wide association studies (GWAS) have identified several risk genes for stroke; however, it remains unclear how they confer risk for the disease. We conducted an integrative analysis to identify candidate genes for stroke and stroke subtypes by integrating blood-derived multi-omics data with genetic data. We systematically integrated the latest stroke GWAS database with human plasma proteomes and performed proteome-wide association studies, Mendelian randomization (MR), Bayesian colocalization analysis and transcriptome-wide association study to prioritize genes that associate the risk of stroke and its subtypes with their expression and protein abundance in plasma. The target genes were verified by performing tissue and cell type specificity, and functional analysis using the Genotype-Tissue Expression database, single-cell RNA sequencing and Gene Ontology databases. A two-step MR analysis was followed to explore the potential mechanisms. We found that the protein abundance of seven genes (*MMP12, F11, SH3BGRL3, ENGASE, SCARA5, SWAP70* and *SPATA20*) in the plasma was associated with stroke and its subtypes, and six genes (*MMP12, F11, SH3BGRL3, SCARA5, SWAP70* and *SPATA20*) causally related with stroke and its subtypes. The effect of *F11, SH3BGRL3, SPATA20* and *SWAP70* on each subtype was mediated by Factor XI inhibitors, atrial fibrillation, type 2 diabetes and systolic blood pressure, respectively (*P* < 0.05). We also found that *SCARA5* and *SWAP70* were related to stroke and ischemic stroke at the transcriptome level. Our present proteomic findings may offer potential future therapeutic targets for stroke prevention.

## Teaser

Liu *et al*. used a multi omics approach to discover and validate a series of genes expressed in plasma that are associated with the risk of stroke and its subtypes.

## Graphical Abstract

**Graphical Abstract:**
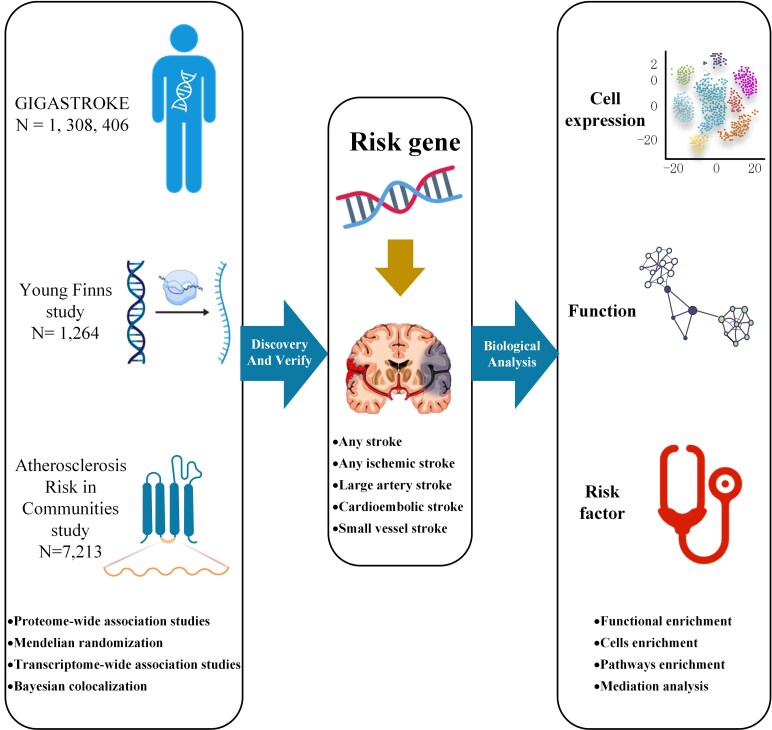

## Introduction

Stroke is one of the leading causes of death and long-term disability worldwide, which imposes a great burden on global health.^1^ Despite significant advancements in the past decades, critical gaps persist in the management of stroke and its subtypes (i.e. large artery atherosclerosis, cardioembolic stroke, or small artery occlusion).^2^ As such, better understanding the mechanism of stroke occurrence, and identifying novel therapeutic targets are needed to tackle this challenge.

In recent years, large-scale genome-wide association studies (GWAS) have significantly advanced the identification of genetic loci associated with stroke and its subtypes.^3^ However, despite advances in understanding the genetic risk factors for stroke, gene expression is regulated at multiple levels, including post-transcriptional, translational and post-translational, which lead to a complex, pleiotropic and polygenic genetic architecture underlying stroke susceptibility. Deciphering the underlying mechanism for these genetic effects on stroke is always challenging, which increases the difficulty in the drug development based on the identified genetic findings.

Proteins represent the ultimate products of gene expression and serve as the primary functional units within cells and biological systems.^4,5^ Directly investigating proteins that are linked to stroke would be of great help in identifying potential therapeutic targets regarding stroke management, especially when this link is supported by the genetic association.^6^ In the past few years, several genetic articles have concerned the role of circulating proteins in stroke, including a proteome-wide association study and one Mendelian randomization (MR) analysis.^7,8^ However, it is likely that the identification of pQTL in their study was incomplete, possibly due to the inadequate sample size or/and lack of comprehensive protein measurements. The multi-omics verification was also lacking. With higher-quality and larger sample sizes of GWAS and, especially, human plasma proteomics data becoming available, to perform a new analysis using the newest data and tools would be promising to identify novel therapeutic targets for stroke.

Accordingly, we performed an integrative analysis to identify candidate genes associated with stroke and its subtypes by combining blood-derived multi-omics datasets with genetic data.

## Materials and methods

### Human plasma proteomic and transcriptomic data

The plasma proteomic data (pQTL) were obtained from a comprehensive analysis of cis-genetic regulation of the plasma proteome, conducted in large European cohorts from the Atherosclerosis Risk in Communities (ARIC) study, comprising curated plasma protein profiles from 7213 European American participants.^9^ Relative concentrations of plasma proteins or protein complexes were measured by the Slow-Of rate Modified Aptamer (SOMAmer) via a proteomic profiling platform.^10,11^ A total of 4657 SOMAmers, which target proteins or protein complexes encoded by 4435 genes, were used to investigate the molecular etiology of stroke. Finally, a total of 2004 proteins were identified.

We obtained the whole-blood eQTL data from the Young Finns Study (YFS). The YFS, which contains 1264 population samples, has been carried out in all five Finnish university cities with medical schools and their rural surroundings. Comprehensive details are available in the original study.^12^ In addition, we used eQTL data from the eQTLGen consortium, which included 31 684 blood samples, for complementary colocalization analysis with the pQTL described above.^13^

### GWAS data of stroke and its subtypes

Genetic association estimates for stroke were derived from the GIGASTROKE consortium, which conducted the largest published stroke GWAS meta-analysis to date.^14^ Populations were restricted to European participants. Data for 1 308 064 European descent individuals (73 652 any stroke cases and 1 234 808 controls) were used as a stroke GWAS summary dataset. The primary outcomes for main analysis were the occurrence of any stroke (including both ischemic and hemorrhagic stroke; AS; 73 652 cases), any ischemic stroke (AIS; 62 100 cases), or three aetiologic ischemic stroke subtypes: Large-artery Atherosclerotic Stroke (LAS; 6399 cases), cardioembolic stroke (CES; 10 804 cases) and small vessel stroke (SVS; 6811 cases).

### GWAS data on risk factors for stroke

Secondary outcomes included established stroke risk factors, identified through a comprehensive review of the literature.^15^ We identified five risk factors and searched for well-powered, publicly available GWAS summary statistics corresponding to them, including systolic blood pressure (SBP),^16^ atrial fibrillation (AF),^17^ type 2 diabetes (T2D),^18^ body mass index (BMI)^19^ and coagulation factor XI (FXI).^20^ Detailed information on the data sources and sample sizes for these GWASs is available in the Supplementary materials (Table S1).

### The RNA-Sequencing data availability of stroke

Single-cell RNA sequencing (scRNA-seq) data were used to identify the cell-type specificity and functional pathways of target genes.^21^ The scRNA-seq data were downloaded from the GEO dataset (https://www.ncbi.nlm.nih.gov/geo/) with accession number GSE174574. This study provided a scRNA-seq landscape of 17 cell populations of mice brain cortex with cell-type specific gene expression profiles based on the middle cerebral artery occlusion (MCAO) model.^21^ Functional pathways of target genes were enriched by Gene ontology (GO) analysis. Cell type-specific expression of the risk genes was displayed with a dot plot according to the cell classification from Zhang *et al*.^21^ The scRNA-seq analysis was performed using R packages of Seurat and ggsci. The enrichment score of risk genes from PWAS was calculated by R package AUCell. All raw data used in this study have been approved for ethical purposes.

### Statistical analysis

#### Proteome-wide and transcriptome-wide association studies

**Figure 1:**
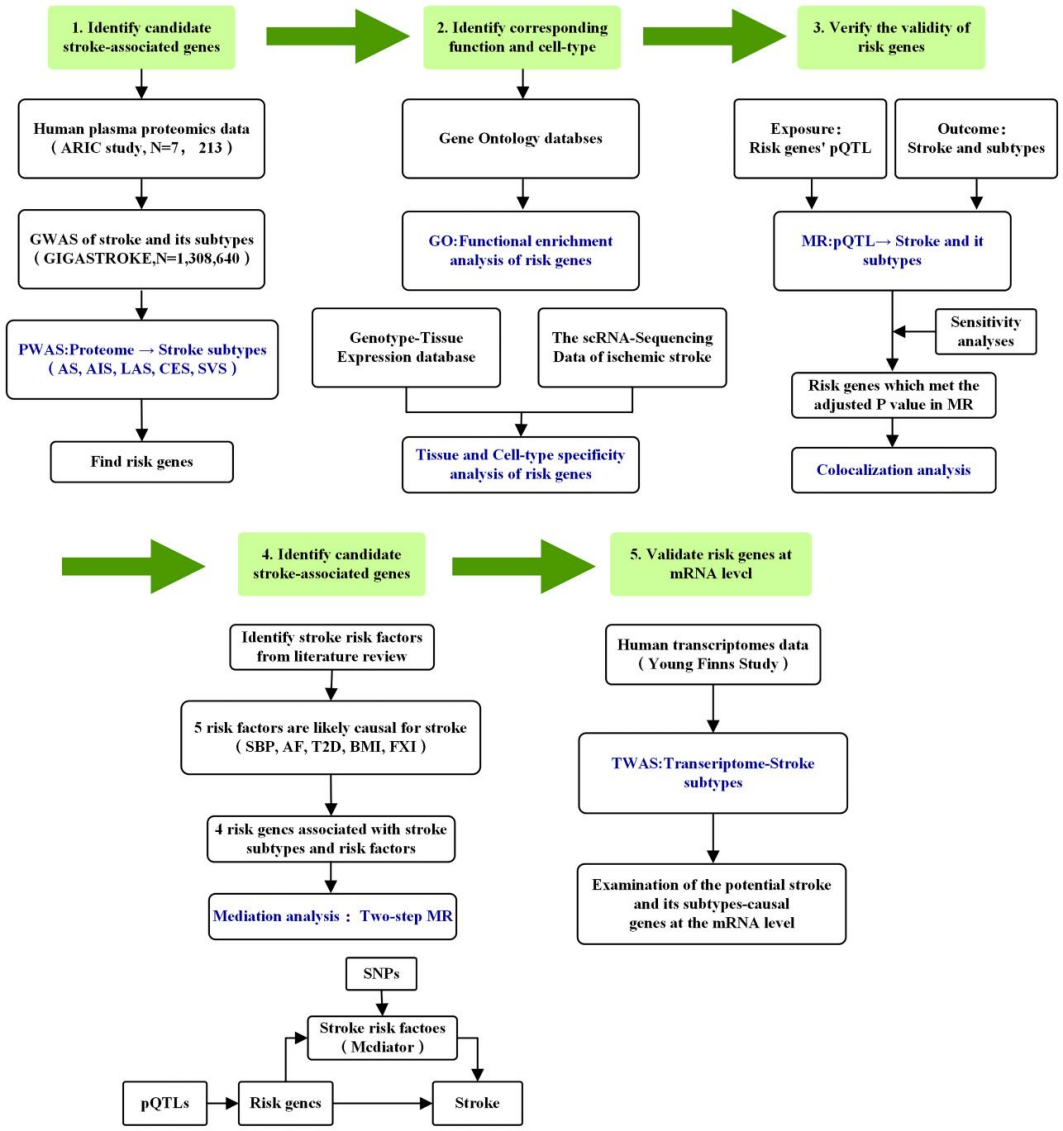
**Overview of this study.** First, we used pQTL datasets obtained from plasma and findings from stroke and its subtypes GWAS to perform a PWAS analysis. Second, we performed cell enrichment and functional enrichment analysis of the identified risk genes using single-cell transcriptomes and GO databases. Third, we used independent MR analysis to verify PWAS-significant genes. Fourth, we used a colocalization to integrate GWAS data and plasma pQTL using a Bayesian colocalization analysis to explore whether two associated signals are consistent with shared causal variant(s). Fifth, we included five common risk factors for stroke in two-step MR analyses to explore the clinical mechanisms of risk genes. Finally, we explored the significant genes driving GWAS signals at the transcriptional level by leveraging gene expression data. ARIC, Atherosclerosis Risk in Communities; GWAS, genome-wide association studies; PWAS, proteome-wide association studies; AS, any stroke; AIS, any ischemic stroke; LAS, large artery stroke; CES, cardioembolic stroke; SVS, small vessel stroke; scRNA-seq, single-cell RNA sequencing; pQTL, protein quantitative trait locus; MR, Mendelian randomization; SBP, systolic blood pressure; AF, atrial fibrillation; T2D, type 2 diabetes; BMI, body mass index; FXI, coagulation factor XI; GTEx, Genotype-Tissue Expression; TWAS, transcriptome-wide association study.

The overall design of this study is shown in Fig. 1. Proteome-wide association studies (PWAS) were carried out using FUSION.^22^ To mitigate the impact of linkage disequilibrium (LD) on the estimated test statistics, an LD reference panel was utilized.^22^ FUSION was used to estimate the effects of single nucleotide polymorphisms (SNPs) on protein abundance for proteins exhibiting significant heritability (*P* < 0.01). Two predictive models, Elastic-net (ENET) and the best single SNP (top1) were used in the analysis.^9^ The ENET model includes multiple SNPs in a multivariable framework, allowing for simultaneous analysis of their combined effects with regularization to avoid overfitting. In contrast, the top1 models analyze one SNP at a time, focusing only on the strongest individual association without considering interactions or correlations with other SNPs. Protein weights from the most predictive model were selected.^9^ The expression weights were derived from human plasma proteomic, and the data were generated from ARIC. Subsequently, the FUSION was used to combine the genetic effect of stroke and stroke subtypes (stroke and its subtypes GWAS z-score) with the protein or expression weights by calculating the linear sum of z-score × weight for the independent SNPs at the locus to perform the PWAS or TWAS. The *P* values were adjusted for false discovery rate (FDR) using the Benjamini–Hochberg (BH) method and the statistical significance was set at *P* < 0.05. Subsequently, using the DrugBank database, we explored specific drugs that target the risk genes obtained by PWAS.^23^

FUSION was also utilized to conduct transcriptome-wide association studies (TWAS).^22^ To determine the optimal gene prediction model, FUSION performed fivefold cross-validation for each model (top1, BLUP, LASSO, ENET and BSLMM) to estimate the out-of-sample R^2^. The imputed gene expression was subsequently used to assess its association with stroke.

### MR analysis

We used two-sample MR to evaluate whether PWAS-significant genes for stroke and its subtypes, identified through FUSION, are associated with these traits via their cis-regulated plasma protein abundance. We also estimated the causal role of proteins encoded by these genes in stroke subtypes and related risk factors. The SNPs included in the study robustly and independently (*r*^2^ < 0.1; clumping window, 10 000 kb) predicted exposures at a genome-wide level (*P* < 5 × 10^−8^). We harmonized the SNP alleles across studies and removed palindromic SNPs with ambiguous allele frequencies (0.42–0.58).

Principal analyses were performed using the inverse-variance weighted (IVW) MR method, which assumes that all genetic variants are valid instrumental variables and provides the most precise causal estimates.^24^ Wald’s ratio method was applied when there was only one SNP available for the target exposure.^25^

The weighted median and MR-Egger regression methods were employed as sensitivity analyses. The weighted median approach assigns greater weight to more precise instrumental variables and yields consistent estimates even when up to 50% of the instruments are invalid or weak.^26^ The MR-Egger could detect and adjust for directional pleiotropy, albeit with low precision.^24^ Cochran’s Q statistic was used within the IVW model to assess heterogeneity across variant-specific estimates. The MR Pleiotropy Residual Sum and Outlier (MR-PRESSO) method was applied to detect potential outlier variants. Finally, a leave-one-SNP-out analysis was performed in which SNPs were systematically removed to assess whether a single SNP drove the results. We calculated the *P* value adjusted for FDR using the BH method. We set the *P* value for statistical significance at *P*-FDR < 0.05. Statistical analyses were conducted in R (version 4.2.2) and MR analyses were conducted using ‘TwoSampleMR’.

### Bayesian colocalization analysis

For each gene associated with one or more stroke outcomes, we conducted colocalization analysis to evaluate whether the genetic associations between protein levels and stroke outcomes were attributable to shared causal variants. Using a multi-trait colocalization approach, we estimated the posterior probability (PP) that multiple traits share the same causal SNP simultaneously.^27^ We used the default colocalization priors of P_1_ = 10^−4^, P_2_ = 10^−4^ and P_12_ = 10^−5^, where P_1_ is the probability that a given variant is associated with stroke or its subtypes, P_2_ is the probability that a given variant is a significant pQTL and P_12_ is the probability that a given variant is both a stroke or its subtypes result and a pQTL. Colocalization uses computed approximation Bayes factors and summary association data generates a PP for the following 5 hypotheses: H_0_, No association with either GWAS or pQTL; H_1_, Association with GWAS, not with pQTL; H_2_, Association with pQTL, not with GWAS; H_3_, Association with GWAS and pQTL, two independent SNPs; and H_4_, Association with GWAS and pQTL, one shared SNP. Our primary focus was the final hypothesis H4, while the PP was utilized to quantify the support for H4, denoted as PPH4. We defined strong evidence of colocalization at PPH4 ≥ 0.75.^28^ In addition, we co-located eQTL and pQTL data for risk genes as a complementary analysis.

### Mediation analysis

For genes whose protein levels were causally associated with both stroke and its risk factors, we conducted mediation analysis to quantify the extent to which protein effects on stroke outcomes were mediated through their influence on risk factors. The total effect of an exposure on an outcome comprises both the direct effect and any indirect effects mediated through one or more intermediate variables. In this study, the total effect is assessed using a standard univariable MR analysis, referred to as the primary MR. To distinguish between direct and indirect effects, we utilized results from a two-step MR approach. The Product method was applied to estimate the beta coefficient of the indirect effect, while the Delta method was employed to determine the standard error (SE) and confidence interval (CI).^29^

### Functional enrichment analysis and cell-type specificity analysis

GO analysis was performed to analyze functional enrichment. Subsequently, we used the Genotype-Tissue Expression (GTEx) database to initially enrich the risk genes at the tissue and cell level in the human body. Finally, we investigated the cell type-specific expression of the risk genes during stroke using mouse single-cell RNA-seq (scRNA-seq) data. The cell suspensions underwent scRNA-seq using standard 10X Chromium Single Cell Chemistry V3. Raw sequencing data were processed using Cellranger to produce gene-level counts for each cell in each sample. The CellRanger mkfastq command was used to generate Fastq files. Data were mapped to a prebuild mouse reference genome. All subsequent analysis was performed using R packages of Seurat and ggsci. The enrichment score of risk genes from PWAS was calculated by R package AUCell. The detailed parameters of scRNA analysis could refer to previous works.^21^

## Results

### PWAS of stroke and its subtypes

**Figure 2:**
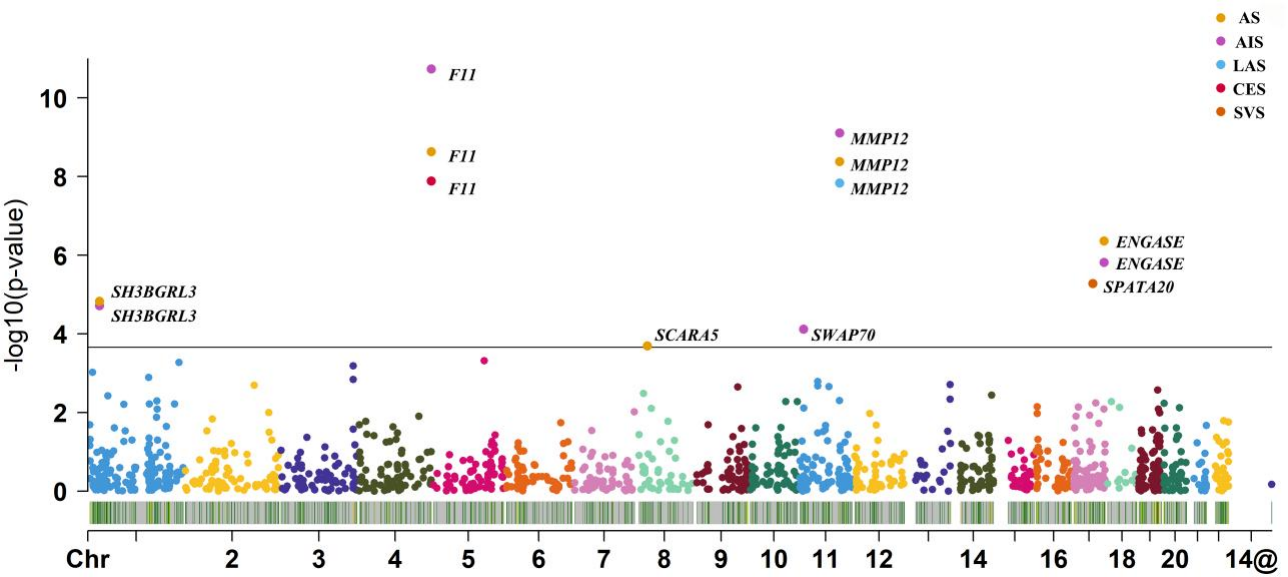
**The Manhattan plot for the PWAS of stroke and stroke subtypes.** The Manhattan plot shows the genes identified using PWAS for AS, AIS, LAS, CES and SVS, respectively. We combined GWAS for stroke and its subtypes (73 652 any stroke cases and 1 234 808 controls) with ARIC proteomic data (*N* = 7 213). Each point represents a single association test between a gene and stroke and its subtypes ordered by genomic position on the x-axis and the association strength on the y-axis as the −log10(P) of a z-score test. The figure includes a total of 1343 data points. The discovery of PWAS identified 7 genes whose cis-regulated plasma protein abundance was associated with stroke subtypes at an FDR of *P* < 0.05. The red horizontal line reflects the significant threshold of the FDR *P* < 0.05. Each data point in the graph represents the correlation between protein abundance of gene expression and stroke risk. The X-axis coordinate represents the specific position of chromosome number. AS: any stroke; AIS: any ischemic stroke; LAS: Large-artery Atherosclerotic Stroke; CES: cardioembolic stroke; SVS: small vessel stroke; Chr, chromosome. ARIC, Atherosclerosis Risk in Communities.

The PWAS identified 7 genes (*MMP12*, *F11*, *ENGASE*, *SH3BGRL3*, *SPATA20*, *SWAP70*, *SCARA5*) whose cis-regulated plasma protein levels were associated with stroke and its subtypes at a FDR of *P* < 0.05 (Table 1, Fig. 2 and Table S2). The protein abundance of *MMP12* was associated with AS, AIS and LAS (AS: Z-score: −5.881, PWAS FDR P = 2.73 × 10^−6^; AIS: Z-score: −6.149, PWAS FDR P = 5.18 × 10^−7^; LAS: Z-score: −5.674, PWAS FDR P = 7.41 × 10^−6^), and the protein abundance of *F11* was associated with AS, AIS and CES (AS: Z-score: 5.972, PWAS FDR P = 3.12 × 10^−6^; AIS: Z-score: 6.718, PWAS FDR P = 2.45 × 10^−8^; CES: Z-score: 5.683, PWAS FDR P = 1.39 × 10^−5^). The protein abundance of *ENGASE* and *SH3BGRL3* were associated with AS and AIS (*ENGASE*: AS: Z-score = 5.055, PWAS FDR P = 1.91 × 10^−4^; AIS: Z-score = 4.805, PWAS FDR P = 6.87 × 10^−4^), (*SH3BGRL3*: AS: Z-score = −4.284, PWAS FDR P = 6.14 × 10^−3^; AIS: Z-score = −4.322, PWAS FDR P = 5.12 × 10^−3^). The protein abundance of *SCARA5*, *SWAP70* and *SPATA20* were associated with AS, AIS and SVS respectively (*SCARA5*: Z-score: −3.712, PWAS FDR P = 4.58 × 10^−2^; *SWAP70*: Z-score: −3.955, PWAS FDR P = 2.03 × 10^−2^; *SPATA20*: Z-score: 4.55, PWAS FDR P = 5.21 × 10^−3^). At present, only two of the seven risk genes have clear targeted drugs (Table 1).

**Table 1:** Summary of seven significant PWAS genes in stroke and its subtypes

Gene	Potential drug	Cell specificity	Stroke subtype	PWAS-pval	MR-pval	PPH4	If replicated in TWAS
MMP12	Neovastat	Microglia	AS	4.1 × 10^−9^	9.79 × 10^−15^	0.99	No profiled
			AIS	7.79 × 10^−10^	6.36 × 10^−16^	0.97	No profiled
			LAS	1.39 × 10^−8^	6.46 × 10^−5^	0.81	No profiled
F11	Coagulation Factor IX (Recombinant)	Fibroblast	AS	2.34 × 10^−9^	8.12 × 10^−8^	0.97	No profiled
			AIS	1.84 × 10^−11^	3.31 × 10^−10^	0.99	No profiled
			CES	1.33 × 10^−8^	5.75 × 10^−11^	0.99	No profiled
ENGASE	N/A	Ependymocyte	AS	4.30 × 10^−7^	4.88 × 10^−7^	0.63	No
			AIS	1.55 × 10^−6^	1.88 × 10^−6^	0.62	No
SH3BGRL3	N/A	Microglia	AS	1.84 × 10^−5^	2.09 × 10^−6^	0.76	No profiled
			AIS	1.54 × 10^−5^	2.01 × 10^−5^	0.93	No profiled
SCARA5	N/A	PVFB	AS	2.06 × 10^−4^	5.63 × 10^−3^	0.77	No
SWAP70	N/A	Endothelial	AIS	7.64 × 10^−5^	1.99 × 10^−3^	0.98	Yes
SPATA20	N/A	N/A	SVS	5.36 × 10^−6^	6.18 × 10^−5^	0.91	Yes

The second column is found in the DrugBank database. In the last column, ‘No profiled’ means that it was not included in the TWAS database, ‘No’ means that the TWAS analysis result was not significant, and ‘Yes’ means PWAS. Both TWAS were significant.

AS, any stroke; AIS, any ischemic stroke; LAS, large artery stroke; CES, cardioembolic stroke; SVS, small vessel stroke; PWAS, proteome-wide association studies; MR, Mendelian randomization; PPH4, posterior probability of H4; TWAS, transcriptome-wide association study.

### Functional enrichment analysis and cell-type specificity analysis

**Figure 3:**
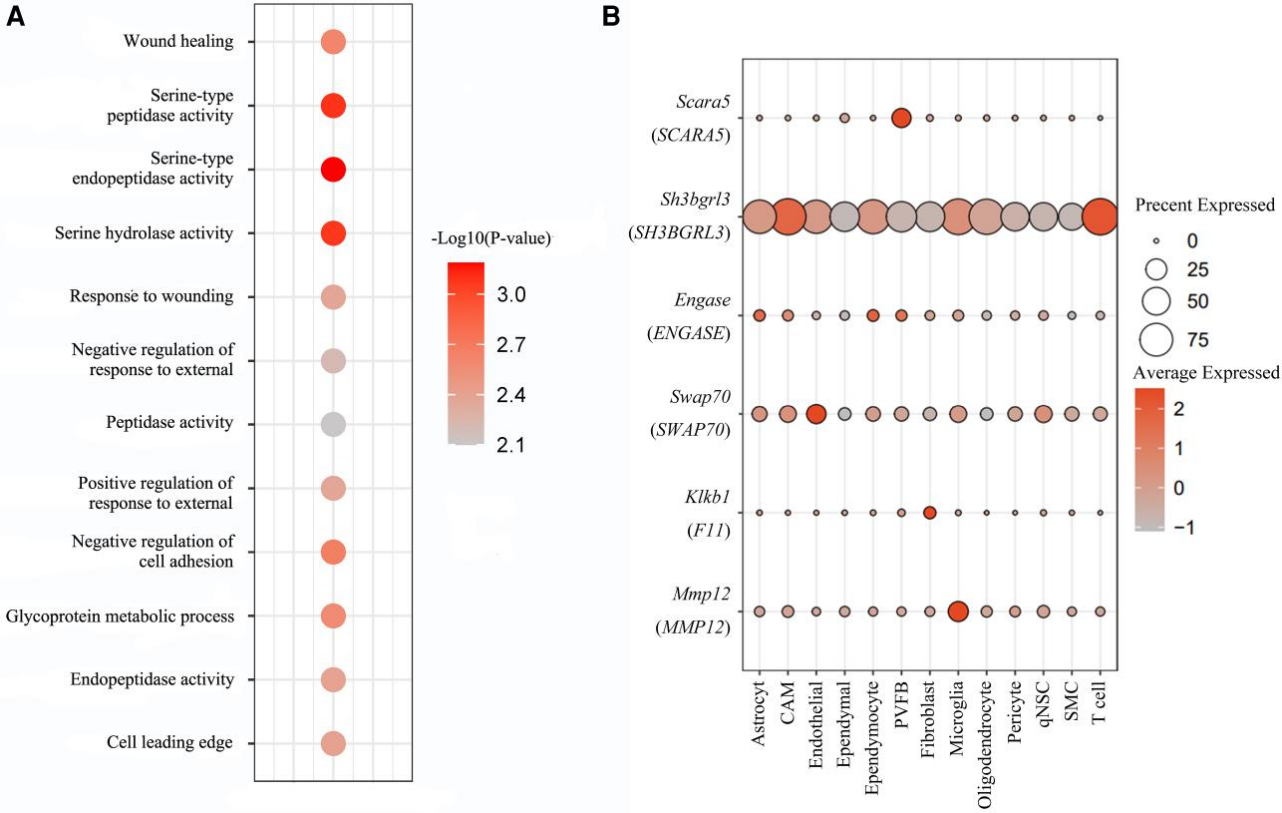
**The functional enrichment analysis and cell-type specificity analysis among candidate genes. (A)** functional enrichment analysis; **(B)** cell-type specificity analysis, single-cell-type expression of the potentially stroke-risk genes. Bar graph of single-cell-type enrichment for risk genes in stroke and its subtypes from the discovery PWAS. The diagram depicts the expression of every gene (*y*-axis) for each cell (*x*-axis), the results of the *P*-values are presented in the Table S4. The single-cell transcriptomic data consisted of the sequencing results of three MCAO mice and three healthy control mice. The size of each data point in Figure A represents the number of genes enriched in this functional pathway. The size of each data point in Figure B represents the average expression percentage of genes in each cell type, while the color depth represents the average expression level. CAM, central nervous system-associated macrophages; PVFB, perivascular fibroblast-like cells; SMC, smooth muscle cells; qNSC, quiescent neural stem cell; MCAO, middle cerebral artery occlusion; PWAS, Proteome-wide association studies.

Then, we investigated whether these risk genes identified by PWAS were enriched in some particular functional pathways and tissue/cell types. Using the GTEx database, we found that these risk genes were highly expressed in human arterial tissues such as the aorta, carotid artery and coronary artery, as well as in whole blood (Fig. S1). At the single-cell level, these risk genes are expressed in fibroblasts and muscle cells in some tissues (Fig. S1). GO analyses suggest that *MMP12* and *F11* were significantly related to serine-type endopeptidase activity, as well as serine-type peptidase activity, wound healing and serine hydrolase activity. *MMP12* and *SWAP70* were significantly related to negative regulation of cell adhesion and positive regulation of response to external stimulus. *MMP12* and *ENGASE* were significantly related to the glycoprotein metabolic process. *SWAP70* and *SH3BGRL3* were significantly related to the cell leading edge (Fig. 3A and Table S3). Finally, we used scRNA-seq data to show different expression patterns of the risk genes in the brain during stroke. *MMP12* was mainly expressed in microglia. *F11* was mainly expressed in fibroblasts. *SCARA5* was mainly expressed in perivascular fibroblast-like cells and *SWAP70* was mainly expressed in endothelial cells. *SH3BGRL3* was abundantly expressed in most cell types of the brain. The expression of *SPATA20* was not detected in this system (Fig. 3B and Table S4).

### Genetically determined PWAS-significant genes and risk of stroke and its subtypes

**Figure 4:**
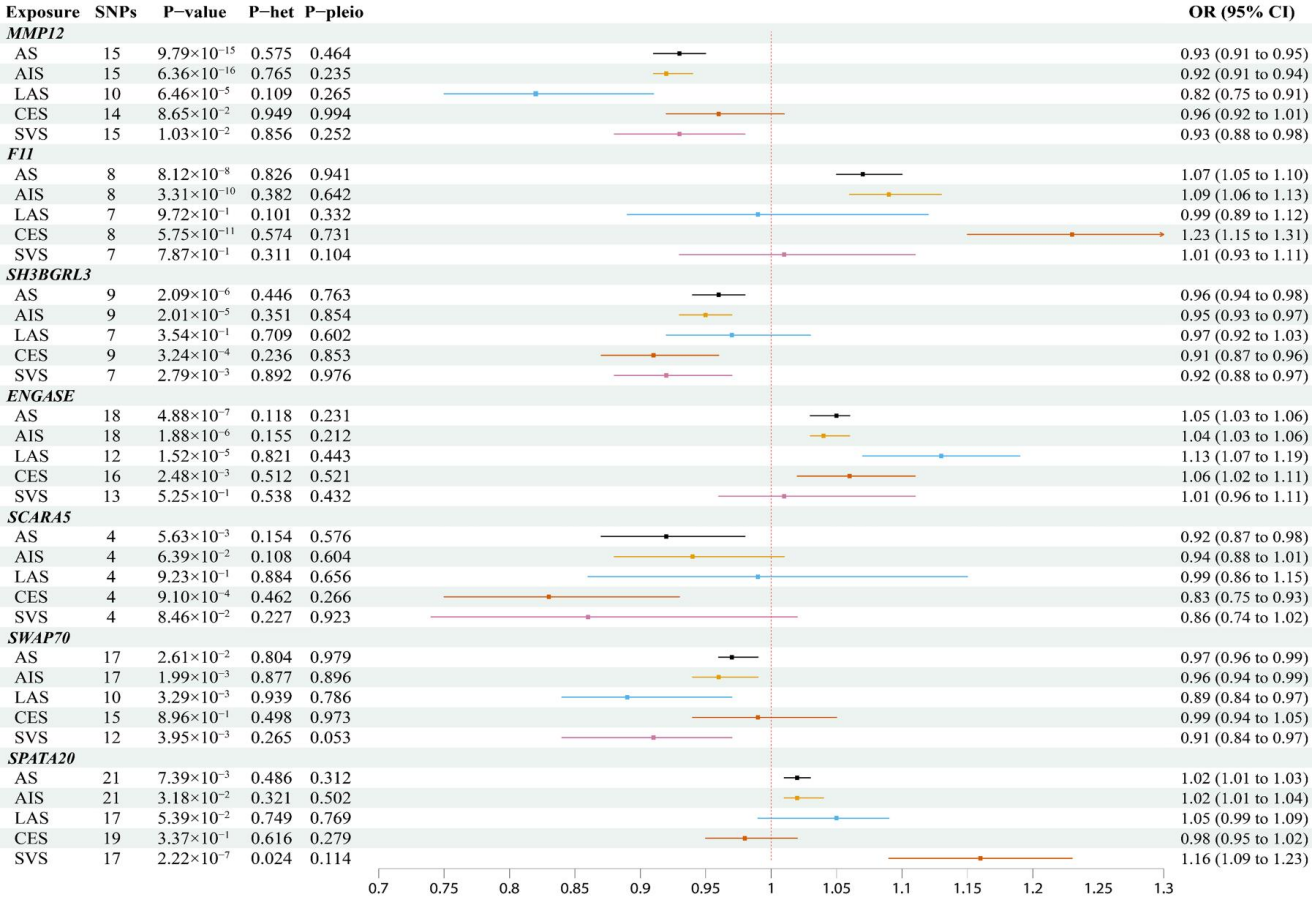
**Effects of stroke and its subtypes PWAS-significant genes on stroke subtypes.** The squares are the causal estimates on the OR scale, and the whiskers represent the 95% CIs for these ORs. SNPs: number of SNPs used for the estimation of the causal effects in this plot. *P*-value was determined from the inverse-variance-weighted two-sample MR method. The *P*-values were adjusted for FDR using the BH method. The data on the *X*-axis represents the numerical value of the odds ratio (OR)P-het, *P* value in the Q statistic for heterogeneity; P-pleio, *P* value in the Egger intercept; AS: any stroke; AIS: any ischemic stroke; LAS: Large-artery Atherosclerotic Stroke; CES: cardioembolic stroke; SVS: small vessel stroke.

Cis-regulated plasma protein levels of seven PWAS-significant genes were tested for causal relationships with stroke and its subtypes by using MR analysis (*P* < 0.007 = 0.05/7). As shown in Fig. 4 and Table 1, we found that the concentration of *MMP12* was inversely associated with AS, AIS and LAS risk (AS: OR [95% CI]: 0.93 [0.91, 0.95], P-FDR = 1.71 × 10^−13^; AIS: OR [95% CI]: 0.92 [0.91, 0.94], P-FDR = 2.23 × 10^−13^; LAS: OR [95% CI]: 0.82 [0.75, 0.91], P-FDR = 1.88 × 10^−4^), while *F11* was positively associated with AS, AIS and CES risk (AS: OR [95% CI]: 1.07 [1.05, 1.10], P-FDR = 5.68 × 10^−7^; AIS: OR [95% CI]: 1.09 [1.06, 1.13], P-FDR = 2.90 × 10^−9^; CES: OR [95% CI]: 1.23 [1.15, 1.31], P-FDR = 6.71 × 10^−10^). The concentration of *ENGASE* was positively associated with AS and AIS (AS: OR [95% CI]: 1.05 [1.03, 1.06], P-FDR = 2.44 × 10^−6^; AIS: OR [95% CI]: 1.04 [1.03, 1.06], P-FDR = 8.23 × 10^−6^), but the concentration of *SH3BGRL3* was inversely associated with AS, AIS (AS: OR [95% CI]: 0.96 [0.94, 0.98], P-FDR = 8.13 × 10^−6^; AIS: OR [95% CI]: 0.95 [0.93, 0.97], P-FDR = 6.40 × 10^−5^). *SCARA5* and *SWAP70* were associated with a lower risk of AS (OR [95% CI]: 0.92 [0.87, 0.98], P-FDR = 9.82 × 10^−3^) and AIS (OR [95% CI]: 0.96 [0.94, 0.99], P-FDR = 4.98 × 10^−3^) while *SPATA20* was associated with a higher risk of SVS (OR [95% CI]: 1.16 [1.09, 1.23], P-FDR = 7.68 × 10^−3^). All sensitivity analyses supported the main results of the MR Analysis (Table S5). The results of MR further confirmed the association between seven genes and their corresponding stroke subtypes.

### Colocalization between stroke risk genes and pQTLs in the plasma

We examined the PP of a shared causal variant between a pQTL and stroke for the seven genes that met the multiple testing–corrected *P*-value threshold in prior PWAS and MR analyses (Table 1). Colocalization analysis suggested that the genetic variants associated with *MMP12* (pQTL) were likely driven by the same causal variants underlying the associations with AS, AIS and LAS (PPH4 ≥ 0.75). Similarly, *F11* pQTLs colocalized with AS, AIS and CES genetic associations; *SH3BGRL3* pQTLs colocalized with AS and AIS; *SCARA5*, *SWAP70* and *SPATA20* pQTLs colocalized with AS, AIS and SVS, respectively. These findings suggest that six of the seven proteins may play important roles in the pathophysiology of stroke subtypes. Interestingly, after collocating the pQTL and eQTL data of risk genes, excluding the two genes that were not included in the eQTL database (*MMP12*, *F11*), we found that the PPH3 of five risk genes was infinitely close to 1 (Table S6). This result may suggest that while both the RNA transcription and protein expression are influenced by the same locus, the underlying regulatory variants of both may be independent. Therefore, this result reflects the complexity of gene regulation, and it can be reasonable.

### Significance of the protein findings

To assess the relevance of six potentially causal genes (excluding ENGASE due to negative results in Bayesian colocalization analysis) identified through PWAS, MR and Bayesian colocalization, we retrieved the lowest *P*-values for SNPs within ±1 Mb of each of the seven genes using summary statistics from the largest stroke GWAS to date (N = 1 308 064).^14^ The most significant *P*-values were below 5 × 10⁻⁸ for two genes (*MMP12* and *F11*), indicating genome-wide significance, while the lowest *P*-values for SNPs in the remaining four genes ranged from 1.32 × 10^−5^ to 2.36 × 10^−6^ (Table S7 and Table 1). These findings suggest that specific plasma proteins may contribute to the pathogenesis of distinct stroke subtypes.

### Mediation effect of six genes on stroke subtypes via risk factors

**Figure 5:**
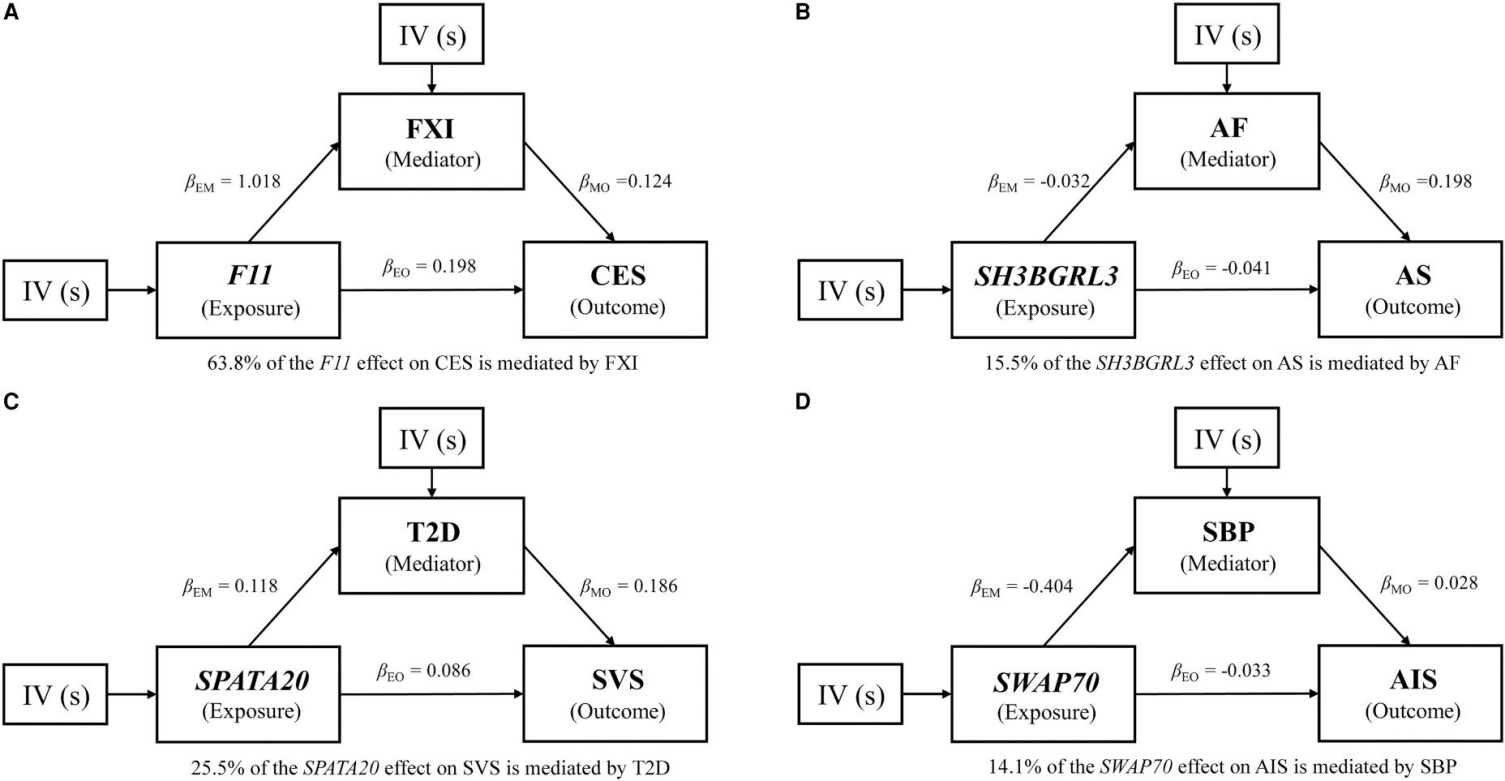
**Mediation effects of genes on stroke via risk factors.** Mediation analyses to quantify the effects of four genes on stroke subtypes via risk factors. **(A)**  *F11* effect on CES mediated by FXI (GWAS-FXI: *N* = 10 708). **(B)**  *SH3BGRL3* effect on AS mediated by AF (GWAS-AF: N-cases = 60, 620). **(C)**  *SPATA20* effect on SVS mediated by T2D (GWAS-T2D: N-cases = 61, 714). **(D)**  *SWAP70* effect on AIS mediated by SBP (GWAS-SBP: N = 757, 601). FXI, coagulation factor XI (eleventh); CES, cardioembolic stroke; SBP, systolic blood pressure; AF, atrial fibrillation; T2D, type 2 diabetes; SVS, small vessel stroke; AS, any stroke; IV, instrumental variable; β_EM_, beta for exposure to mediator; β_EO_, beta for exposure to outcome; β_MO_, beta for mediator to outcome; AIS, any ischemic stroke.

To investigate the indirect effects of cis-regulated plasma protein levels of six genes on stroke subtypes through risk factors, we performed mediation analysis using effect estimates from two-step MR and total effects derived from the primary MR analysis. We calculated the causal relationship between five risk factors and stroke using MR Analysis (Table S7). This analysis was restricted to four genes, *F11*, *SWAP70*, *SH3BGRL3* and *SPATA20*, which showed evidence of an effect in both MRs with risk factors and stroke outcomes (Tables S8–S9). The mediation effect of *F11* via FXI is the highest (63.8%). The indirect effect of *SPATA20* on the risk of SVS via T2D contributes to one-fourth of the total effect (25.2%). Similarly, the proportion of the mediation effect of *SWAP70* and *SH3BGRL3* on AIS and AS via SBP and AF is one-tenth, respectively (*SWAP70*: 14.1%; *SH3BGRL3*: 15.5%) (Fig. 5 and Table S10).

### Examination of the potential stroke-causal proteins at the mRNA level

We integrated GWAS data for stroke subtypes with human plasma transcriptomic data to perform TWAS for stroke and its subtypes using FUSION. We found that the cis-regulated plasma mRNA expression of the forty-one genes was associated with stroke and its subtypes (FDR *P* < 0.05) (Table S11). Interestingly, two genes identified in TWAS (*SWAP70* and *SPATA20*) were also significant in the AIS and SVS PWAS, respectively (Table S12), suggesting joint evidence from PWAS and TWAS for its role in stroke etiology.

## Discussion

In the present study, we employed an integrated analysis based on GWAS, PWAS, TWAS, MR and Bayesian colocalization to investigate the causal relationship between proteins in the plasma and stroke and its subtypes. We identified 7 potential risk genes (*MMP12, F11, SH3BGRL3, ENGASE, SCARA5, SWAP70* and *SPATA20*) of stroke with altered protein abundances in the plasma. The summary results of 7 potential risk genes are shown in Table 1. We enriched all seven genes into corresponding functional pathways and cell types. Six (*MMP12, F11, SH3BGRL3, SCARA5, SWAP70* and *SPATA20*) of the above genes were demonstrated in the MR and Bayesian colocalization analysis validation analyses of stroke and its subtypes, providing a higher confidence level. Furthermore, *SWAP70* and *SPATA20* were supported at the transcriptional level. Through a mediation analysis, we found the effect of *F11* on CES, *SH3BGRL3* on AS and AIS, *SPATA20* on SVS and *SWAP70* on AIS were partially mediated by FXI, AF, T2D and SBP, respectively, demonstrating a critical role of these risk factors in the pathogenesis of stroke in consistence with previous epidemiological studies.^30-34^

Of the 7 genes studied, *MMP12*, *F11* and *SCARA5* have been implicated in previous articles.^7,8^  *MMP12* is a member of the matrix metalloproteinase family that contributes to vascular remodeling. To date, the relationship between its circulating level and stroke is unclear and contradicted in previous studies. In one population-based study containing 2983 participants, higher *MMP12* level was independently associated with ischemic stroke risk (HR = 1.30, 95% CI 1.16–1.45, *P* = 4.55 × 10^−06^).^35^ In contrast, our study is consistent with another genetic study which showed an inverse relationship between *MMP12* circulating level and risk of ischemic stroke.^36^ FXI is mediated by *F11* and was recently proved to be intensively related to the pathogenesis of thrombosis. A GWAS analysis containing 371 695 participants found a lower FXI level was associated with reduced risks of venous thrombosis (OR = 0.1, 95%CI 0.07–0.14; P = 3 × 10^−43^) and ischemic stroke (OR = 0.47, 0.36–0.61; P = 2 × 10^−8^).^37^ MR analysis of our study also revealed a causal relationship between *F11* and CES, with 63.8% of the effect mediated by FXI. Currently, FXI inhibitors have been developed as potential stroke targets, and their role is evaluated by some ongoing trials.^38-40^  *SCARA5* is a scavenger receptor that exports ferritin-bound iron from circulation to parenchymal tissues, including the heart and brain.^41^ Previous MR studies revealed that *SCARA5* can genetically lower the level of serum iron and further decrease CES risk.^8,42^ Using multi-omics analysis, we confirmed this result, that *SCARA5* was associated with a lower risk of AS (OR [95% CI]: 0.92 [0.87, 0.98]).

The discovery of risk genes for stroke and its subtypes has a strong potential for developing novel blood biomarkers. These biomarkers could improve risk stratification, allowing earlier detection and targeted prevention for high-risk individuals. They could also enhance diagnostic accuracy by distinguishing among stroke subtypes, leading to more tailored treatment strategies. Additionally, these genetic markers may provide prognostic insights, helping to predict stroke outcomes and guide personalized care. Future studies are needed to validate these findings and integrate them into clinical practice for improved stroke management.

To our knowledge, the other four genes including *ENGASE*, *SPATA20*, *SWAP70* and *SH3BGRL3* have not been extensively studied in stroke research. We were able to identify a shared causal variant between a pQTL and stroke for the four genes except for *ENGASE*. We found that *SPATA20* might be associated with the SVS subtype. Moreover, mediation analysis of the effects of these three genes on stroke subtypes indicated potential mechanisms. For example, the indirect effect of *SPATA20* on the risk of SVS via T2D contributes to about one-fourth (25.2%) of the total effect. The proportion of the mediation effect of *SWAP70* and *SH3BGRL3* on AIS and AS via SBP and AF is exceeded one-tenth, respectively (*SWAP70*: 14.1%; *SH3BGRL3*: 15.2%). However, the mechanisms of the internal effects served by these proteins are not clear yet. *ENGASE* is an enzyme involved in the processing of free oligosaccharides in the cytosol and is associated with glycosylation. Studies have shown protein glycosylation that may affect the occurrence of AIS by regulating the progression of atherosclerosis and AF.^43^  *SWAP70* is a guanine nucleotide exchange factor that participates in the regulation of many cellular processes, but its role in many diseases has not yet been clarified. However, studies showed that *SWAP70* is a protective molecule that can suppress the progression of nonalcoholic fatty liver disease by inhibiting hepatic steatosis and inflammation^44^ and Pathological Cardiac Hypertrophy,^45^ both of these pathological procedures were associated with stroke occurrence. *SWAP70* also organizes the actin cytoskeleton, which is crucial for phagocytosis.^46^ The role of *SWAP70* in immune function may be related to its protective effect against AIS. *SH3BGRL3* may play a key role in maintaining cerebrovascular integrity and maintaining the function of cerebral vascular endothelial cells by activating STAT3 signaling, thereby preventing AS and AIS.^47,48^  *SPATA20* was predicted to be located in the extracellular region, and involved in the carbohydrate metabolic process and cell differentiation. However, further studies are needed to confirm the role of these genes and proteins in stroke.

The strengths of our study include the utilization of the largest and most comprehensive human proteome from the most recent GWAS and pQTL datasets and integrative analysis of multi-omics data including GWAS, PWAS and TWAS. The stroke GWAS summary datasets we used comprising data for 1 308 064 European descent individuals, nearly three times the size of previous studies (MRGASTROKE *n* = 446, 696).^7,8^ The plasma proteomic data we used including a total of 7 213 European Americans, and the number of significant SOMAmers in this study is almost three times that of plasma pQTL studies that conducted in the past in the European ancestry sample.^49^ These large sample data might increase the comprehensive and representative property of our study. Compared to OLINKS and Somascan, SOMAmers are designed to disengage very slowly from target proteins, which means they can form highly stable complexes, and SOMAmers can cover a wider range of proteins.

Our study also has some limitations. First, although the plasma proteomic data we used included SOMAmers for approximately 5000 proteins or protein complexes, it does not provide coverage for the entire plasma proteome.^9^ Also, the data did not explore the effects of uncommon and rare variants, as well as complex trans-associations, analysis with substantial discovery on an even larger sample size was likely to be needed. Second, it is insufficient to elucidate the numerous stroke PWAS-identified loci from genetic and transcriptional levels. Functional genomic approaches are needed to identify the complex molecular mechanisms of stroke. Third, our study mainly focused on European subjects, and caution should be taken when generalizing our results to other ethnicities. Finally, there are subtle but detectable genetic differences between European Americans and continental Europeans that may have slightly skewed the final results.

## Conclusion

In conclusion, we found causal evidence supporting three classic genes *MMP12*, *F11* and *SCARA5,* and three novel genes *SH3BGRL3*, *SWAP70* and *SPATA20* were associated with the risk of ischemic stroke and their subtypes. Our findings provide genetic evidence underlying ischemic stroke and subtypes, allowing novel therapeutic targets to be further identified.

## Supplementary Material

fcaf457_Supplementary_Data

## Data Availability

All data used in the study came from public databases, and details can be found in the source literature.
