# Supplementary material for: Identification of potential therapeutic targets for stroke and its subtypes by integrating proteomes and genetics from human plasma
Source: Brain Commun. 2025 Dec 30;8(1):fcaf457. doi: 10.1093/braincomms/fcaf457 (PMC12758125; doi:10.1093/braincomms/fcaf457)
Supplement: fcaf457_Supplementary_Data [file fcaf457_supplementary_data.docx]

**Supplementary Online Material**

Table S1. Information on GWAS data sources.

Table S2. The discovery of stroke and its subtypes PWAS identified 7 significant genes.

Table S3. The result of Functional enrichment analysis

Table S4. Single-cell-type expression of the potentially stroke-risk genes

Table S5. Sensitivity analyses for the Mendelian randomization associations between risk genes and stroke subtypes.

Table S6. Results of colocalization of risk genes eQTL and pQTL.

Table S7. SNPs located within 1 Mb of each of the risk genes with the lowest p-value for association with stroke and its subtypes.

Table S8. MR results of risk factors versus stroke and its subtypes.

Table S9. MR results of risk genes and stroke risk factors.

Table S10. MR mediation results for 4 protein targets on stroke outcomes via risk factors.

Table S11. The TWAS of stroke integrates the stroke and its subtypes GWAS with Young Finns Study database transcriptome and genetic data using FUSION.

Table S12. The stroke TWAS verified 2 significant genes.

Figure S1. RNA and protein expression across tissue and cell types for stroke-associated proteins

Reference List

**Table S1.** Information on data sources.

| **Data source** | **Variable** | **cases** | **controls** | **Ancestry** |
| --- | --- | --- | --- | --- |
| GIGASTROKE consortium^1^ | Any stroke | 73,652 | 1,234,808 | European |
|  | Ischemic stroke | 62,100 | 1,234,808 | European |
|  | Large-artery Atherosclerotic Stroke | 6,399 | 1,234,808 | European |
|  | Cardioembolic stroke | 10,804 | 1,234,808 | European |
|  | Small vessel stroke | 6,811 | 1,234,808 | European |
| ICBP + UKBB^2^ | Systolic blood pressure | 757,601 | NA | European |
| UKBB and AFGen consortium et.al^3^ | Atrial fibrillation | 60,620 | 970,216 | European |
| DIAGRAM + GERA + UKBB^4^ | Type 2 diabetes | 61,714 | 597,602 | European |
| GIANT consortium^5^ | Body mass index | 681,275 | NA | European |
| KORA F4 + QMDiab^6^ | Coagulation factor XI | NA | NA | European |

Detailed descriptions of the corresponding datasets are available in the original publications, which are provided in the Supplementary References List.

ICBP: International Consortium of Blood Pressure-Genome Wide Association Studies; UKBB: UK Biobank; DIAGRAM: DIAbetes Genetics Replication and Meta-analysis

**Table S2.** The discovery of stroke and its subtypes PWAS identified 7 significant genes.

| **Subtypes** | **Gene** | **CHR** | **pQTL.ID** | **pQTL.R^2^** | **NSNP** | **MODEL** | **MODELCV.R^2^** | **PWAS.Z** | **PWAS.P** | **PWAS.FDR.P** |
| --- | --- | --- | --- | --- | --- | --- | --- | --- | --- | --- |
| AS | F11 | 4 | rs2289252 | 0.083288 | 78 | enet | 0.11 | 5.97204 | 2.34E-09 | 3.12E-06 |
| AS | MMP12 | 11 | rs2276109 | 0.124634 | 66 | enet | 0.15 | -5.88018 | 4.10E-09 | 2.73E-06 |
| AS | ENGASE | 17 | rs56107536 | 0.107 | 53 | enet | 0.27 | 5.0551 | 4.30E-07 | 1.91E-04 |
| AS | SH3BGRL3 | 1 | rs4659423 | 0.181 | 61 | enet | 0.21 | -4.2839 | 1.84E-05 | 6.14E-03 |
| AS | SCARA5 | 8 | rs2685422 | 0.024653 | 74 | enet | 0.035 | -3.7116 | 2.06E-04 | 4.58E-02 |
| AIS | F11 | 4 | rs2289252 | 0.083288 | 78 | enet | 0.11 | 6.71797 | 1.84E-11 | 2.45E-08 |
| AIS | MMP12 | 11 | rs2276109 | 0.124634 | 66 | enet | 0.15 | -6.1492 | 7.79E-10 | 5.18E-07 |
| AIS | ENGASE | 17 | rs56107536 | 0.107 | 53 | enet | 0.27 | 4.8049 | 1.55E-06 | 6.87E-04 |
| AIS | SH3BGRL3 | 1 | rs4659423 | 0.181 | 61 | enet | 0.21 | -4.32218 | 1.54E-05 | 5.12E-03 |
| AIS | SWAP70 | 11 | rs10770059 | 0.171952 | 58 | enet | 0.2 | -3.9554 | 7.64E-05 | 2.03E-02 |
| LAS | MMP12 | 11 | rs2276109 | 0.124634 | 66 | enet | 0.15 | -5.674413 | 1.39E-08 | 7.4087E-06 |
| CES | F11 | 4 | rs2289252 | 0.083288 | 78 | enet | 0.11 | 5.6826 | 1.33E-08 | 1.39E-05 |
| SVS | SPATA20 | 17 | rs9890200 | 0.212866 | 40 | top1 | 0.21 | 4.55 | 5.36E-06 | 5.21E-03 |

AS, any stroke; AIS, any ischemic stroke; LAS, Large-artery Atherosclerotic Stroke; CES, cardioembolic stroke; SVS, small vessel stroke; pQTL.R^2^, cross-validation R^2^ of the best pQTL in the locus; MODEL, Best performing model; MODELCV.R^2^, cross-validation R^2^ of the best performing model;

**Table S3.** The result of Functional enrichment analysis.

| **GroupID** | **Category** | **Term** | **Description** | **LogP** | **Symbols** |
| --- | --- | --- | --- | --- | --- |
| 1_Summary | GO Molecular Functions | GO:0004252 | serine-type endopeptidase activity | -3.18699 | F11,MMP12 |
| 1_Member | GO Molecular Functions | GO:0004252 | serine-type endopeptidase activity | -3.18699 | F11,MMP12 |
| 1_Member | GO Molecular Functions | GO:0008236 | serine-type peptidase activity | -3.09107 | F11,MMP12 |
| 1_Member | GO Molecular Functions | GO:0017171 | serine hydrolase activity | -3.07312 | F11,MMP12 |
| 1_Member | GO Biological Processes | GO:0042060 | wound healing | -2.61473 | F11,MMP12 |
| 1_Member | GO Molecular Functions | GO:0004175 | endopeptidase activity | -2.39587 | F11,MMP12 |
| 1_Member | GO Biological Processes | GO:0009611 | response to wounding | -2.36664 | F11,MMP12 |
| 1_Member | GO Biological Processes | GO:0032102 | negative regulation of response to external stimulus | -2.36664 | F11,MMP12 |
| 1_Member | GO Molecular Functions | GO:0008233 | peptidase activity | -2.09658 | F11,MMP12 |
| 2_Summary | GO Biological Processes | GO:0007162 | negative regulation of cell adhesion | -2.66524 | MMP12,SWAP70 |
| 2_Member | GO Biological Processes | GO:0007162 | negative regulation of cell adhesion | -2.66524 | MMP12,SWAP70 |
| 2_Member | GO Biological Processes | GO:0032103 | positive regulation of response to external stimulus | -2.21175 | MMP12,SWAP70 |
| 3_Summary | GO Biological Processes | GO:0009100 | glycoprotein metabolic process | -2.57444 | MMP12,ENGASE |
| 3_Member | GO Biological Processes | GO:0009100 | glycoprotein metabolic process | -2.57444 | MMP12,ENGASE |
| 4_Summary | GO Cellular Components | GO:0031252 | cell leading edge | -2.40786 | SWAP70,SH3BGRL3 |
| 4_Member | GO Cellular Components | GO:0031252 | cell leading edge | -2.40786 | SWAP70,SH3BGRL3 |

**Table S4.** Single-cell-type expression of the potentially stroke-risk genes.

|  | **Mmp12** | **Klkb1** | **Swap70** | **Engase** | **Sh3bgrl3** | **Scara5** |
| --- | --- | --- | --- | --- | --- | --- |
| Astrocyte | 1.29E-01 | 3.01E-03 | 4.27E-01 | 1.75E-01 | 7.35E+00 | 1.05E-03 |
| CAM | 1.98E-01 | 6.32E-04 | 4.53E-01 | 1.16E-01 | 1.49E+01 | 1.34E-03 |
| Endothelial | 6.26E-02 | 6.09E-04 | 9.99E-01 | 5.11E-02 | 7.24E+00 | 5.51E-03 |
| Ependymal | 7.39E-02 | 8.24E-04 | 1.27E-01 | 3.89E-02 | 2.12E+00 | 3.19E-02 |
| Ependymocyte | 8.95E-02 | 4.72E-03 | 3.63E-01 | 1.87E-01 | 7.66E+00 | 1.25E-03 |
| PVFB | 6.11E-02 | 1.71E-02 | 3.05E-01 | 1.58E-01 | 3.02E+00 | 9.35E-01 |
| Fibroblast | 8.68E-02 | 1.62E-01 | 2.06E-01 | 7.39E-02 | 2.89E+00 | 1.17E-02 |
| Microglia | 4.51E+00 | 2.59E-03 | 3.88E-01 | 7.92E-02 | 8.82E+00 | 4.32E-03 |
| Oligodendrocyte | 1.12E-01 | 0.00E+00 | 1.22E-01 | 4.43E-02 | 5.72E+00 | 4.15E-03 |
| Pericyte | 4.70E-01 | 0.00E+00 | 3.12E-01 | 6.00E-02 | 3.51E+00 | 2.37E-03 |
| qNSC | 2.00E-01 | 3.48E-03 | 4.63E-01 | 6.81E-02 | 2.91E+00 | 2.75E-03 |
| SMC | 6.53E-02 | 4.94E-04 | 2.82E-01 | 3.05E-02 | 2.39E+00 | 1.11E-03 |
| Tcell | 8.96E-02 | 0.00E+00 | 2.86E-01 | 4.51E-02 | 1.72E+01 | 0.00E+00 |

CAM, central nervous system (CNS)-associated macrophages; FB, perivascular fibroblast-like cells; SMC, vascular smooth muscle cells.

**Table S5.** Sensitivity analyses for the Mendelian randomization associations between risk genes and stroke subtypes.

| **Exposure** | **Outcome** | **Method** | **OR** | **95%CI** | **P** |
| --- | --- | --- | --- | --- | --- |
| MMP12 | AS | IVW (primary analysis) | 0.93 | (0.91-0.95) | 9.79E-15 |
|  |  | MR- egger | 0.94 | (0.91-0.97) | 1.53E-03 |
|  |  | Weighted median | 0.94 | (0.92-0.96) | 2.88E-07 |
|  |  | Egger Intercept | -0.003 | N/A | 4.64E-01 |
|  | AIS | IVW (primary analysis) | 0.92 | (0.91-0.94) | 6.36E-15 |
|  |  | MR- egger | 0.94 | (0.91-0.97) | 2.18E-03 |
|  |  | Weighted median | 0.93 | (0.91-0.96) | 1.45E-07 |
|  |  | Egger Intercept | -0.005 | N/A | 2.35E-01 |
|  | LAS | IVW (primary analysis) | 0.82 | (0.75-0.91) | 6.46E-05 |
|  |  | MR- egger | 0.76 | (0.66-0.89) | 8.87E-03 |
|  |  | Weighted median | 0.83 | (0.77-0.89) | 5.63E-07 |
|  |  | Egger Intercept | 0.026 | N/A | 2.65E-01 |
|  | CES | IVW (primary analysis) | 0.96 | (0.92-1.01) | 8.65E-02 |
|  |  | MR- egger | 0.96 | (0.89-1.04) | 3.26E-01 |
|  |  | Weighted median | 0.96 | (0.91-1.01) | 1.52E-01 |
|  |  | Egger Intercept | 8.17E-05 | N/A | 9.94E-01 |
|  | SVS | IVW (primary analysis) | 0.93 | (0.88-0.98) | 1.03E-02 |
|  |  | MR- egger | 0.97 | (0.88-1.07) | 6.11E-01 |
|  |  | Weighted median | 0.96 | (0.89-1.03) | 2.05E-01 |
|  |  | Egger Intercept | -0.017 | N/A | 2.52E-01 |
| F11 | AS | IVW (primary analysis) | 1.07 | (1.05-1.10) | 8.12E-08 |
|  |  | MR- egger | 1.07 | (1.01-1.13) | 5.43E-02 |
|  |  | Weighted median | 1.07 | (1.05-1.10) | 5.95E-07 |
|  |  | Egger Intercept | 5.82E-04 | NA | 9.41E-01 |
|  | AIS | IVW (primary analysis) | 1.09 | (1.06-1.13) | 3.31E-10 |
|  |  | MR- egger | 1.11 | (1.04-1.19) | 1.98E-02 |
|  |  | Weighted median | 1.1 | (1.07-1.14) | 6.18E-09 |
|  |  | Egger Intercept | -0.004 | NA | 6.42E-01 |
|  | LAS | IVW (primary analysis) | 0.99 | (0.89-1.12) | 9.72E-01 |
|  |  | MR- egger | 1.12 | (0.88-1.43) | 3.94E-01 |
|  |  | Weighted median | 1.02 | (0.93-1.13) | 6.78E-01 |
|  |  | Egger Intercept | -0.034 | NA | 3.32E-01 |
|  | CES | IVW (primary analysis) | 1.23 | (1.16-1.31) | 5.75E-11 |
|  |  | MR- egger | 1.21 | (1.05-1.38) | 3.59E-02 |
|  |  | Weighted median | 1.24 | (1.15-1.34) | 1.91E-08 |
|  |  | Egger Intercept | 0.007 | NA | 7.31E-01 |
|  | SVS | IVW (primary analysis) | 1.01 | (0.93-1.10) | 7.87E-01 |
|  |  | MR- egger | 1.18 | (0.99-1.39) | 1.17E-01 |
|  |  | Weighted median | 1.04 | (0.95-1.14) | 3.71E-01 |
|  |  | Egger Intercept | -0.046 | NA | 1.04E-01 |
| SH3BGRL3 | AS | IVW (primary analysis) | 0.96 | (0.94-0.98) | 2.09E-06 |
|  |  | MR- egger | 0.96 | (0.94-0.99) | 2.81E-02 |
|  |  | Weighted median | 0.95 | (0.94-0.98) | 2.53E-05 |
|  |  | Egger Intercept | -0.002 | NA | 7.63E-01 |
|  | AIS | IVW (primary analysis) | 0.96 | (0.94-0.98) | 2.01E-05 |
|  |  | MR- egger | 0.96 | (0.93-0.99) | 2.98E-02 |
|  |  | Weighted median | 0.95 | (0.94-0.97) | 8.15E-06 |
|  |  | Egger Intercept | 0.001 | NA | 8.54E-01 |
|  | LAS | IVW (primary analysis) | 0.97 | (0.92-1.03) | 3.54E-01 |
|  |  | MR- egger | 0.98 | (0.89-1.08) | 6.89E-01 |
|  |  | Weighted median | 0.98 | (0.92-1.04) | 4.41E-01 |
|  |  | Egger Intercept | -0.003 | NA | 6.02E-01 |
|  | CES | IVW (primary analysis) | 0.91 | (0.87-0.96) | 3.24E-04 |
|  |  | MR- egger | 0.93 | (0.86-1.01) | 9.79E-02 |
|  |  | Weighted median | 0.93 | (0.89-0.98) | 4.11E-03 |
|  |  | Egger Intercept | 0.004 | NA | 8.53E-01 |
|  | SVS | IVW (primary analysis) | 0.92 | (0.88-0.97) | 2.79E-03 |
|  |  | MR- egger | 0.92 | (0.85-1.00) | 9.76E-02 |
|  |  | Weighted median | 0.93 | (0.87-0.98) | 8.84E-03 |
|  |  | Egger Intercept | 0.0004 | NA | 9.76E-01 |
| ENGASE | AS | IVW (primary analysis) | 1.05 | (1.03-1.06) | 4.88E-07 |
|  |  | MR- egger | 1.06 | (1.03-1.09) | 8.98E-04 |
|  |  | Weighted median | 1.05 | (1.03-1.07) | 3.57E-07 |
|  |  | Egger Intercept | -0.006 | NA | 2.31E-01 |
|  | AIS | IVW (primary analysis) | 1.05 | (1.03-1.06) | 1.88E-06 |
|  |  | MR- egger | 1.06 | (1.03-1.09) | 1.14E-03 |
|  |  | Weighted median | 1.05 | (1.03-1.08) | 2.84E-06 |
|  |  | Egger Intercept | -0.007 | NA | 2.12E-01 |
|  | LAS | IVW (primary analysis) | 1.13 | (1.07-1.19) | 1.52E-05 |
|  |  | MR- egger | 1.17 | (1.06-1.28) | 9.11E-03 |
|  |  | Weighted median | 1.13 | (1.05-1.21) | 6.06E-04 |
|  |  | Egger Intercept | -0.012 | NA | 4.43E-01 |
|  | CES | IVW (primary analysis) | 1.06 | (1.02-1.11) | 2.48E-03 |
|  |  | MR- egger | 1.04 | (0.98-1.11) | 2.18E-01 |
|  |  | Weighted median | 1.06 | (1.01-1.11) | 3.02E-02 |
|  |  | Egger Intercept | 0.007 | NA | 5.21E-01 |
|  | SVS | IVW (primary analysis) | 1.02 | (0.97-1.07) | 5.25E-01 |
|  |  | MR- egger | 1.05 | (0.96-1.14) | 3.24E-01 |
|  |  | Weighted median | 1.04 | (0.98-1.11) | 2.16E-01 |
|  |  | Egger Intercept | -0.01 | NA | 4.32E-01 |
| SCARA5 | AS | IVW (primary analysis) | 0.92 | (0.87-0.98) | 5.33E-03 |
|  |  | MR- egger | 0.87 | (0.73-1.05) | 2.79E-01 |
|  |  | Weighted median | 0.92 | (0.88-0.97) | 2.61E-03 |
|  |  | Egger Intercept | 0.01 | N/A | 5.76E-01 |
|  | AIS | IVW (primary analysis) | 0.94 | （0.88-1.01） | 6.39E-02 |
|  |  | MR- egger | 0.88 | （0.72-1.09） | 3.62E-01 |
|  |  | Weighted median | 0.94 | （0.88-0.99） | 2.77E-02 |
|  |  | Egger Intercept | 0.011 | N/A | 6.04E-01 |
|  | LAS | IVW (primary analysis) | 0.99 | (0.86-1.15) | 9.23E-01 |
|  |  | MR- egger | 0.91 | (0.61-1.34) | 6.56E-01 |
|  |  | Weighted median | 0.97 | (0.83-1.14) | 7.18E-01 |
|  |  | Egger Intercept | 0.017 | N/A | 6.56E-01 |
|  | CES | IVW (primary analysis) | 0.83 | (0.75-0.93) | 9.10E-01 |
|  |  | MR- egger | 0.67 | (0.50-0.90) | 1.19E-01 |
|  |  | Weighted median | 0.83 | (0.73-0.94) | 3.53E-03 |
|  |  | Egger Intercept | 0.039 | N/A | 2.66E-01 |
|  | SVS | IVW (primary analysis) | 0.86 | (0.73-1.02) | 8.46E-02 |
|  |  | MR- egger | 0.89 | (0.51-1.54) | 7.18E-01 |
|  |  | Weighted median | 0.86 | (0.94-1.02) | 7.91E-02 |
|  |  | Egger Intercept | -0.005 | N/A | 9.23E-01 |
| SWAP70 | AS | IVW (primary analysis) | 0.98 | (0.96-0.99) | 2.61E-02 |
|  |  | MR- egger | 0.97 | (0.92-1.04) | 5.22E-01 |
|  |  | Weighted median | 0.98 | (0.96-1.01) | 1.85E-01 |
|  |  | Egger Intercept | -0.001 | N/A | 9.79E-01 |
|  | AIS | IVW (primary analysis) | 0.97 | (0.94-0.99) | 1.99E-03 |
|  |  | MR- egger | 0.97 | (0.91-1.04) | 4.19E-01 |
|  |  | Weighted median | 0.98 | (0.95-1.01) | 1.66E-01 |
|  |  | Egger Intercept | -0.001 | N/A | 8.96E-01 |
|  | LAS | IVW (primary analysis) | 0.89 | (0.84-0.97) | 3.29E-03 |
|  |  | MR- egger | 0.93 | (0.74-1.17) | 5.44E-01 |
|  |  | Weighted median | 0.91 | (0.83-0.99) | 3.43E-02 |
|  |  | Egger Intercept | -0.007 | N/A | 7.86E-01 |
|  | CES | IVW (primary analysis) | 0.99 | (0.95-1.05) | 8.96E-01 |
|  |  | MR- egger | 0.99 | (0.85-1.17) | 9.93E-01 |
|  |  | Weighted median | 0.98 | (0.91-1.05) | 5.54E-01 |
|  |  | Egger Intercept | -0.001 | N/A | 9.73E-01 |
|  | SVS | IVW (primary analysis) | 0.91 | (0.84-0.97) | 3.95E-03 |
|  |  | MR- egger | 1.13 | (0.91-1.40) | 2.88E-01 |
|  |  | Weighted median | 0.92 | (0.84-1.01) | 7.64E-02 |
|  |  | Egger Intercept | -0.055 | N/A | 5.30E-02 |
| SPATA20 | AS | IVW (primary analysis) | 1.02 | (1.00-1.03) | 7.39E-03 |
|  |  | MR- egger | 1.03 | (1.00-1.06) | 2.96E-02 |
|  |  | Weighted median | 1.03 | (1.01-1.04) | 8.08E-03 |
|  |  | Egger Intercept | -0.004 | N/A | 3.12E-01 |
|  | AIS | IVW (primary analysis) | 1.02 | (1.00-1.03) | 3.18E-02 |
|  |  | MR- egger | 1.03 | (0.99-1.06) | 9.74E-02 |
|  |  | Weighted median | 1.02 | (0.99-1.04) | 5.59E-02 |
|  |  | Egger Intercept | -0.003 | N/A | 5.02E-01 |
|  | LAS | IVW (primary analysis) | 1.05 | (0.99-1.10) | 5.39E-02 |
|  |  | MR- egger | 1.04 | (0.95-1.12) | 4.17E-01 |
|  |  | Weighted median | 1.03 | (0.97-1.09) | 3.38E-01 |
|  |  | Egger Intercept | 0.004 | N/A | 7.69E-01 |
|  | CES | IVW (primary analysis) | 0.98 | (0.95-1.02) | 3.37E-01 |
|  |  | MR- egger | 1.01 | (0.95-1.08) | 7.01E-01 |
|  |  | Weighted median | 0.99 | (0.95-1.04) | 7.53E-01 |
|  |  | Egger Intercept | -0.011 | N/A | 2.79E-01 |
|  | SVS | IVW (primary analysis) | 1.16 | (1.09-1.23) | 2.22E-07 |
|  |  | MR- egger | 1.21 | (1.09-1.33) | 1.91E-03 |
|  |  | Weighted median | 1.13 | (1.06-1.19) | 6.18E-05 |
|  |  | Egger Intercept | -0.025 | N/A | 1.14E-01 |

AS, any stroke; AIS, any ischemic stroke; LAS, Large-artery Atherosclerotic Stroke; CES, cardioembolic stroke; SVS, small vessel stroke; IVW, Inverse variance weighted.

**Table S6.** Results of colocalization of risk genes eQTL and pQTL.

| **Gene** | **PPH0** | **PPH1** | **PPH2** | **PPH3** | **PPH4** |
| --- | --- | --- | --- | --- | --- |
| SPATA20 | 0 | 0 | 0 | 9.12E-01 | 8.77E-02 |
| SCARA5 | 0 | 0 | 0 | 9.99E-01 | 4.92E-04 |
| SH3BGRL3 | 0 | 0 | 9.60E-48 | 1 | 2.15E-20 |
| SWAP70 | 0 | 0 | 1.61E-257 | 1 | 1.30E-61 |
| ENGASE | 0 | 1.59E-179 | 0 | 1 | 2.05E-47 |

**Table S7.** SNPs located within 1 Mb of each of the risk genes with the lowest p-value for association with stroke and its subtypes.

| **Outcome** | **Gene** | **Chr** | **SNP with lowest p-value** | **SNP p-value** |
| --- | --- | --- | --- | --- |
| AS | *F11* | 4 | rs6843711 | 9.05E-08 |
| AS | *MMP12* | 11 | rs150986675 | 1.08E-09 |
| AS | *SH3BGRL3* | 1 | rs4659423 | 1.32E-05 |
| AS | *SCARA5* | 8 | rs10866867 | 1.36E-05 |
| AIS | *F11* | 4 | rs3756011 | 6.31E-10 |
| AIS | *MMP12* | 11 | rs150986675 | 3.40E-10 |
| AIS | *SWAP70* | 11 | rs34716494 | 2.36E-06 |
| AIS | *SH3BGRL3* | 1 | rs11247922 | 8.06E-06 |
| LAS | *MMP12* | 11 | rs476762 | 3.42E-08 |
| CES | *F11* | 4 | rs4253399 | 6.30E-08 |
| SVS | *SPATA20* | 17 | rs9890200 | 5.37E-06 |

AS, any stroke; AIS, any ischemic stroke; LAS, Large-artery Atherosclerotic Stroke; CES, cardioembolic stroke; SVS, small vessel stroke; SNP, single nucleotide polymorphism.

**Table S8.** MR results of risk factors versus stroke and its subtypes.

| **Exposure** | **Outcome** | **Method** | **Beta** | **OR** | **P** |
| --- | --- | --- | --- | --- | --- |
| SBP | AS | IVW (primary analysis) | 0.0268 | 1.03 | 2.86E-48 |
|  |  | MR- egger | 0.0336 | 1.03 | 1.08E-11 |
|  |  | Weighted median | 0.0271 | 1.03 | 6.81E-31 |
|  |  | Egger Intercept | -0.002 | N/A | 1.26E-01 |
|  | AIS | IVW (primary analysis) | 0.0276 | 1.03 | 9.72E-44 |
|  |  | MR- egger | 0.0346 | 1.04 | 8.21E-11 |
|  |  | Weighted median | 0.0281 | 1.03 | 3.99E-29 |
|  |  | Egger Intercept | 0.001 | N/A | 1.41E-01 |
|  | LAS | IVW (primary analysis) | 0.054 | 1.06 | 1.64E-29 |
|  |  | MR- egger | 0.059 | 1.06 | 5.16E-06 |
|  |  | Weighted median | 0.045 | 1.05 | 1.35E-10 |
|  |  | Egger Intercept | -0.002 | N/A | 6.78E-01 |
|  | CES | IVW (primary analysis) | 0.0228 | 1.02 | 1.27E-09 |
|  |  | MR- egger | 0.0349 | 1.04 | 4.35E-04 |
|  |  | Weighted median | 0.0305 | 1.03 | 1.44E-08 |
|  |  | Egger Intercept | -0.004 | N/A | 1.82E-01 |
|  | SVS | IVW (primary analysis) | 0.0408 | 1.04 | 5.37E-18 |
|  |  | MR- egger | 0.038 | 1.04 | 2.29E-03 |
|  |  | Weighted median | 0.0452 | 1.05 | 3.44E-11 |
|  |  | Egger Intercept | 0.001 | N/A | 8.41E-01 |
| AF | AS | IVW (primary analysis) | 0.157 | 1.17 | 7.28E-30 |
|  |  | MR- egger | 0.127 | 1.14 | 4.28E-06 |
|  |  | Weighted median | 0.175 | 1.19 | 9.13E-24 |
|  |  | Egger Intercept | 0.003 | N/A | 1.83E-01 |
|  | AIS | IVW (primary analysis) | 0.164 | 1.18 | 4.77E-30 |
|  |  | MR- egger | 0.136 | 1.15 | 3.05E-06 |
|  |  | Weighted median | 0.177 | 1.19 | 5.54E-19 |
|  |  | Egger Intercept | 0.003 | N/A | 2.17E-01 |
|  | LAS | IVW (primary analysis) | 0.007 | 1.01 | 8.61E-01 |
|  |  | MR- egger | -0.55 | 0.94 | 4.71E-01 |
|  |  | Weighted median | 0.037 | 1.04 | 5.57E-01 |
|  |  | Egger Intercept | 0.005 | N/A | 3.39E-01 |
|  | CES | IVW (primary analysis) | 0.644 | 1.91 | 1.41E-141 |
|  |  | MR- egger | 0.743 | 2.11 | 1.09E-27 |
|  |  | Weighted median | 0.675 | 1.97 | 5.64E-64 |
|  |  | Egger Intercept | -0.009 | N/A | 1.58E-01 |
|  | SVS | IVW (primary analysis) | 0.036 | 1.04 | 3.01E-01 |
|  |  | MR- egger | -0.051 | 0.95 | 4.47E-01 |
|  |  | Weighted median | 0.057 | 1.01 | 8.99E-01 |
|  |  | Egger Intercept | 0.008 | N/A | 1.26E-01 |
| T2D | AS | IVW (primary analysis) | 0.077 | 1.08 | 1.42E-09 |
|  |  | MR- egger | 0.025 | 1.03 | 4.09E-01 |
|  |  | Weighted median | 0.049 | 1.05 | 3.01E-03 |
|  |  | Egger Intercept | 0.004 | N/A | 6.50E-02 |
|  | AIS | IVW (primary analysis) | 0.088 | 1.09 | 2.71E-10 |
|  |  | MR- egger | 0.039 | 1.04 | 2.48E-01 |
|  |  | Weighted median | 0.072 | 1.07 | 1.38E-04 |
|  |  | Egger Intercept | 0.004 | N/A | 1.15E-01 |
|  | LAS | IVW (primary analysis) | 0.158 | 1.17 | 1.11E-04 |
|  |  | MR- egger | 0.159 | 1.17 | 1.42E-01 |
|  |  | Weighted median | 0.153 | 1.16 | 7.56E-03 |
|  |  | Egger Intercept | -0.0001 | N/A | 9.87E-01 |
|  | CES | IVW (primary analysis) | 0.066 | 1.07 | 1.31E-02 |
|  |  | MR- egger | 0.021 | 1.02 | 7.61E-01 |
|  |  | Weighted median | 0.065 | 1.07 | 1.08E-01 |
|  |  | Egger Intercept | 0.003 | N/A | 4.71E-01 |
|  | SVS | IVW (primary analysis) | 0.142 | 1.15 | 6.59E-05 |
|  |  | MR- egger | 0.007 | 1.01 | 9.49E-01 |
|  |  | Weighted median | 0.134 | 1.14 | 1.12E-02 |
|  |  | Egger Intercept | 0.009 | N/A | 1.86E-01 |
| BMI | AS | IVW (primary analysis) | 0.151 | 1.16 | 4.05E-09 |
|  |  | MR- egger | 0.167 | 1.18 | 1.25E-02 |
|  |  | Weighted median | 0.131 | 1.14 | 7.49E-04 |
|  |  | Egger Intercept | -0.0002 | N/A | 7.91E-01 |
|  | AIS | IVW (primary analysis) | 0.165 | 1.18 | 1.88E-09 |
|  |  | MR- egger | 0.195 | 1.21 | 6.78E-03 |
|  |  | Weighted median | 0.146 | 1.16 | 8.26E-04 |
|  |  | Egger Intercept | -0.0005 | N/A | 6.57E-01 |
|  | LAS | IVW (primary analysis) | 0.241 | 1.27 | 3.09E-03 |
|  |  | MR- egger | 0.181 | 1.19 | 3.97E-01 |
|  |  | Weighted median | 0.134 | 1.14 | 3.01E-01 |
|  |  | Egger Intercept | 0.001 | N/A | 7.62E-01 |
|  | CES | IVW (primary analysis) | 0.136 | 1.15 | 1.97E-02 |
|  |  | MR- egger | 0.289 | 1.34 | 5.69E-02 |
|  |  | Weighted median | 0.069 | 1.07 | 4.53E-01 |
|  |  | Egger Intercept | -0.003 | N/A | 2.73E-01 |
|  | SVS | IVW (primary analysis) | 0.215 | 1.24 | 3.31E-03 |
|  |  | MR- egger | 0.193 | 1.21 | 3.28E-01 |
|  |  | Weighted median | 0.121 | 1.18 | 1.69E-01 |
|  |  | Egger Intercept | 0.0004 | N/A | 8.83E-01 |
| FXI | AS | IVW (primary analysis) | 0.056 | 1.06 | 3.62E-05 |
|  |  | MR- egger | 0.083 | 1.09 | 1.81E-01 |
|  |  | Weighted median | 0.067 | 1.07 | 3.84E-12 |
|  |  | Egger Intercept | -0.012 | N/A | 5.95E-01 |
|  | AIS | IVW (primary analysis) | 0.071 | 1.07 | 1.79E-06 |
|  |  | MR- egger | 0.071 | 1.07 | 2.92E-01 |
|  |  | Weighted median | 0.073 | 1.08 | 8.19E-12 |
|  |  | Egger Intercept | 0.001 | N/A | 9.92E-01 |
|  | LAS | IVW (primary analysis) | 0.03 | 1.03 | 2.22E-01 |
|  |  | MR- egger | 0.147 | 1.16 | 1.58E-01 |
|  |  | Weighted median | 0.041 | 1.04 | 1.78E-01 |
|  |  | Egger Intercept | -0.053 | N/A | 2.15E-01 |
|  | CES | IVW (primary analysis) | 0.134 | 1.14 | 2.66E-06 |
|  |  | MR- egger | 0.121 | 1.13 | 3.39E-01 |
|  |  | Weighted median | 0.119 | 1.13 | 8.35E-06 |
|  |  | Egger Intercept | 0.006 | N/A | 9.02E-01 |
|  | SVS | IVW (primary analysis) | 0.069 | 1.07 | 2.57E-03 |
|  |  | MR- egger | 0.061 | 1.06 | 4.61E-01 |
|  |  | Weighted median | 0.069 | 1.07 | 7.94E-03 |
|  |  | Egger Intercept | 0.003 | N/A | 9.27E-01 |

AS, any stroke; AIS, any ischemic stroke; LAS, Large-artery Atherosclerotic Stroke; CES, cardioembolic stroke; SVS, small vessel stroke; IVW, Inverse variance weighted, SBP, systolic blood pressure; AF, atrial fibrillation; T2D, type 2 diabetes; BMI, body mass index; FXI, coagulation factor XI.

**Table S9.** MR results of risk genes and stroke risk factors.

| **Exposure** | **Outcome** | **Method** | **Beta** | **OR** | **P** |
| --- | --- | --- | --- | --- | --- |
| F11 | FXI | IVW (primary analysis) | 1.018 | 2.77 | 7.56E-29 |
|  |  | MR- egger | 1.186 | 3.27 | 1.22E-01 |
|  |  | Weighted median | 1.016 | 2.76 | 1.04E-23 |
| SH3BGRL3 | AF | IVW (primary analysis) | -0.032 | 0.96 | 1.30E-04 |
|  |  | MR- egger | -0.048 | 0.95 | 5.02E-03 |
|  |  | Weighted median | -0.035 | 0.97 | 2.84E-04 |
| SPATA20 | T2D | IVW (primary analysis) | 0.118 | 1.13 | 1.05E-02 |
|  |  | MR- egger | 0.075 | 1.01 | 9.32E-01 |
|  |  | Weighted median | 0.066 | 1.07 | 1.27E-02 |
| SWAP70 | SBP | IVW (primary analysis) | -0.404 | 0.67 | 5.36E-04 |
|  |  | MR- egger | -0.861 | 0.42 | 3.34E-03 |
|  |  | Weighted median | -0.486 | 0.62 | 1.00E-15 |

SBP, systolic blood pressure; AF, atrial fibrillation; T2D, type 2 diabetes; FXI, coagulation factor XI; IVW, Inverse variance weighted.

**Table S10.** MR mediation results for 4 protein targets on stroke outcomes via risk factors.

| **Exposure** | **Mediator** | **Outcome** | **Mediation/Indirect effect** | | **Total Effect** | | | **proportion of mediation effect** |
| --- | --- | --- | --- | --- | --- | --- | --- | --- |
|  |  |  | **beta** | **se** | **beta** | **se** | **pval** |  |
| F11 | FXI | Cardioembolic-stroke | 0.126 | 0.091 | 0.198 | 0.037 | 1.21E-07 | 63.80% |
| SH3BGRL3 | AF | Any stroke | -0.006 | 0.004 | -0.041 | 0.008 | 2.09E-06 | 15.50% |
| SPATA20 | T2D | small vessel stroke | 0.022 | 0.009 | -0.086 | 0.02 | 2.23E-07 | 25.20% |
| SWAP70 | SBP | Any ischemic stroke | 0.011 | 0.004 | -0.033 | 0.01 | 1.99E-03 | 14.10% |

**Table S11.** The TWAS of stroke integrates the stroke and its subtypes GWAS with Young Finns Study database transcriptome and genetic data using FUSION.

| **Outcome** | **Gene** | **CHR** | **EQTL.R2** | **EQTL.Z** | **MODEL** | **TWAS.Z** | **TWAS.P** | **TWAS.FDR.P** |
| --- | --- | --- | --- | --- | --- | --- | --- | --- |
| AS | *SH2B3* | 12 | 0.00891 | -5.61 | enet | 5.66049 | 1.51E-08 | 7.08E-05 |
| AS | *ALDH2* | 12 | 0.0708 | 9.69 | enet | -5.06979 | 3.98E-07 | 9.34E-04 |
| AS | *EIF6* | 20 | 0.003773 | 4.58 | blup | -4.70247 | 2.57E-06 | 4.02E-03 |
| AS | *NUP133* | 1 | 0.002415 | -4.28 | bslmm | -4.679 | 2.88E-06 | 3.38E-03 |
| AS | *SREBF1* | 17 | 0.112871 | -12.2 | top1 | -4.456 | 8.35E-06 | 7.84E-03 |
| AS | *TMEM106B* | 7 | 0.055 | 8.73 | lasso | 4.40167 | 1.07E-05 | 8.37E-03 |
| AS | *TRPC4AP* | 20 | 0.157887 | 14.47 | bslmm | 4.37238 | 1.23E-05 | 8.24E-03 |
| AS | *TM6SF1* | 15 | 0.072051 | 9.75 | bslmm | 4.32805 | 1.50E-05 | 8.80E-03 |
| AS | *ANXA3* | 4 | 0.0455 | 8.1 | bslmm | 4.2535 | 2.10E-05 | 1.09E-02 |
| AS | *GATAD1* | 7 | 0.00565 | -4.69 | blup | 4.20776 | 2.58E-05 | 1.21E-02 |
| AS | *UBE2G1* | 17 | 0.01219 | -4.83 | top1 | -4.137 | 3.52E-05 | 1.50E-02 |
| AS | *CENPQ* | 6 | 0.0067 | 4.81 | blup | -4.06129 | 4.88E-05 | 1.91E-02 |
| AS | *ACAD10* | 12 | 0.00846 | 3.75 | bslmm | -4.00765 | 6.13E-05 | 2.21E-02 |
| AS | *ITFG3* | 16 | 0.0762 | -10.12 | bslmm | 3.97985 | 6.90E-05 | 2.31E-02 |
| AS | *CTNNBL1* | 20 | 4.58E-04 | -4.24 | enet | 3.97189 | 7.13E-05 | 2.23E-02 |
| AS | *ROMO1* | 20 | 0.15002 | 14.72 | enet | -3.95137 | 7.77E-05 | 2.28E-02 |
| AS | *DUSP16* | 12 | 0.00842 | -5.13 | enet | -3.92027 | 8.84E-05 | 2.44E-02 |
| AS | *ATXN2* | 12 | 0.0186 | 6.58 | blup | -3.87501 | 1.07E-04 | 2.79E-02 |
| AS | *PROCR* | 20 | 0.001423 | -3.98 | blup | 3.86896 | 1.09E-04 | 2.69E-02 |
| AS | *SEMA4D* | 9 | 0.204816 | 16.23 | enet | 3.86231 | 1.12E-04 | 2.63E-02 |
| AS | *NFE2* | 12 | 0.0159 | 5.06 | bslmm | -3.82011 | 1.33E-04 | 2.97E-02 |
| AS | *LARS* | 5 | 0.223 | -16.82 | top1 | 3.716 | 2.02E-04 | 4.31E-02 |
| AIS | *SH2B3* | 12 | 0.00891 | -5.61 | enet | 6.58818 | 4.45E-11 | 2.09E-07 |
| AIS | *ALDH2* | 12 | 0.0708 | 9.69 | enet | -5.35614 | 8.50E-08 | 1.99E-04 |
| AIS | *TRPC4AP* | 20 | 0.157887 | 14.47 | bslmm | 4.9272 | 8.34E-07 | 1.30E-03 |
| AIS | *EIF6* | 20 | 0.003773 | 4.58 | blup | -4.6788 | 2.88E-06 | 3.38E-03 |
| AIS | *ACAD10* | 12 | 0.00846 | 3.75 | bslmm | -4.53191 | 5.85E-06 | 5.49E-03 |
| AIS | *ATXN2* | 12 | 0.0186 | 6.58 | blup | -4.38868 | 1.14E-05 | 8.91E-03 |
| AIS | *TMEM106B* | 7 | 0.055 | 8.73 | lasso | 4.37779 | 1.20E-05 | 8.04E-03 |
| AIS | *UBE2G1* | 17 | 0.01219 | -4.83 | top1 | -4.137 | 3.52E-05 | 2.06E-02 |
| AIS | *NUP133* | 1 | 0.002415 | -4.28 | bslmm | -4.12471 | 3.71E-05 | 1.93E-02 |
| AIS | *SREBF1* | 17 | 0.112871 | -12.2 | top1 | -4.068 | 4.74E-05 | 2.22E-02 |
| AIS | *CENPQ* | 6 | 0.0067 | 4.81 | blup | -3.96719 | 7.27E-05 | 3.10E-02 |
| AIS | *GATAD1* | 7 | 0.00565 | -4.69 | blup | 3.95283 | 7.72E-05 | 3.02E-02 |
| AIS | *ROMO1* | 20 | 0.15002 | 14.72 | enet | -3.9154 | 9.02E-05 | 3.26E-02 |
| AIS | *CTNNBL1* | 20 | 4.58E-04 | -4.24 | enet | 3.9146 | 9.06E-05 | 3.04E-02 |
| AIS | *STXBP5* | 6 | 0.0396 | 7.82 | lasso | 3.87851 | 1.05E-04 | 3.28E-02 |
| AIS | *ADORA3* | 1 | -3.79E-04 | 3.7 | enet | -3.78017 | 1.57E-04 | 4.60E-02 |
| AIS | *ANXA3* | 4 | 0.0455 | 8.1 | bslmm | 3.7602 | 1.70E-04 | 4.69E-02 |
| SVS | *CSTF3* | 11 | 0.01814 | 6.05 | bslmm | -4.76972 | 1.84E-06 | 8.63E-03 |
| SVS | *SPATA20* | 17 | 0.707489 | -29.89 | bslmm | 4.44569 | 8.76E-06 | 2.05E-02 |

AS, any stroke; AIS, any ischemic stroke; SVS, small vessel stroke

**Table S12.** The stroke TWAS verified 2 significant genes.

| **Outcome** | **Gene** | **CHR** | **EQTL.R2** | **EQTL.Z** | **MODEL** | **TWAS.P** | **TWAS.FDR.P** |
| --- | --- | --- | --- | --- | --- | --- | --- |
| SVS | *SPATA20* | 17 | 0.707489 | -29.89 | bslmm | 8.76E-06 | 2.05E-02 |
| AIS | *SWAP70* | 11 | 0.072046 | 9.69 | bslmm | 3.28E-03 | 1.71E-01 |

AIS, any ischemic stroke; SVS, small vessel stroke;


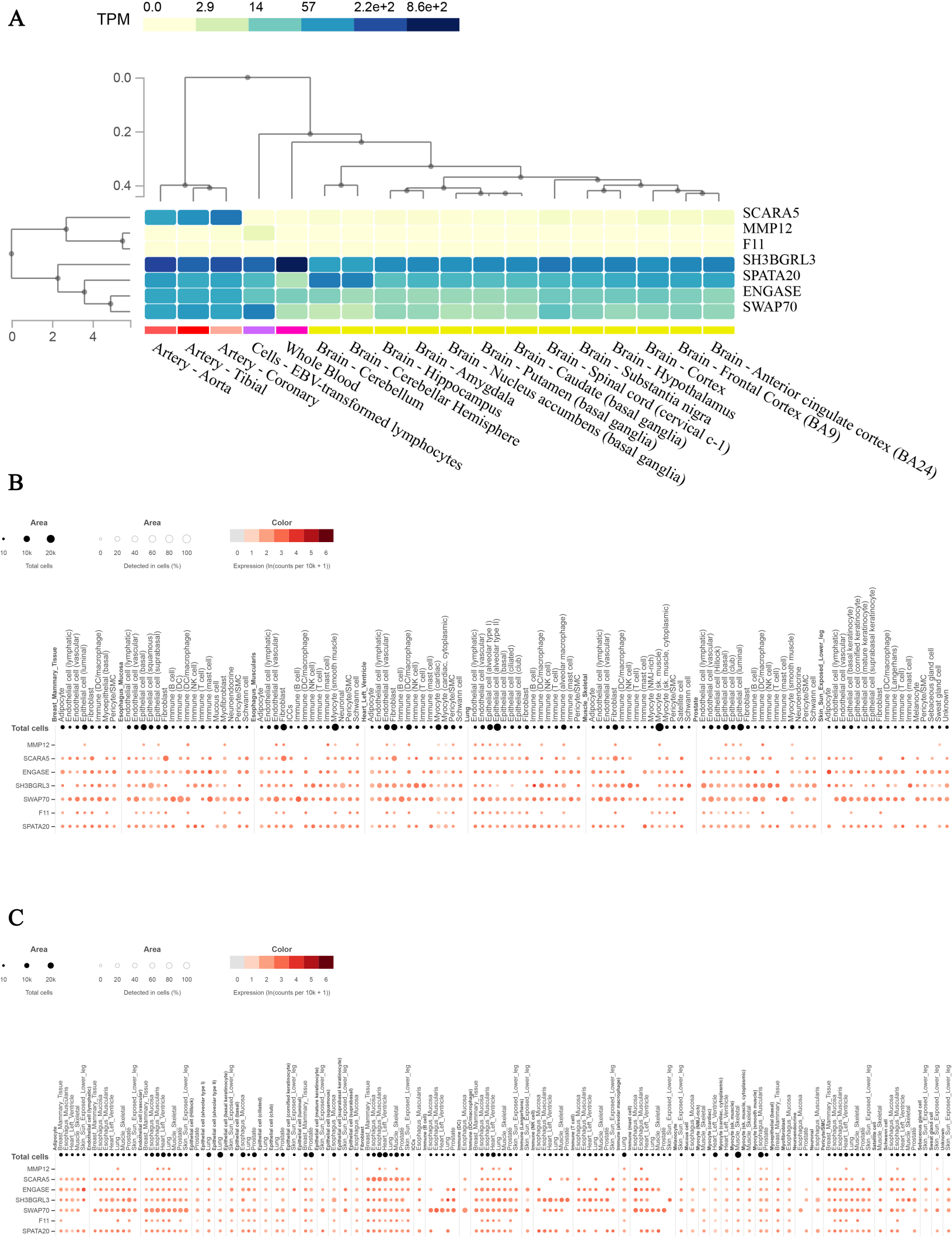


**Figure S1.** RNA and protein expression across tissue and cell types for stroke-associated proteins

1. Gene expression in brain, whole blood, and artery using data available from samples in the GTEx databases; The expression of genes coding for stroke-associated proteins is displayed in transcripts per million. Genes and tissues are grouped on the y- and x-axis based using hierarchical clustering; **B.** Gene expression in brain and other tissue using data available GTEx (X-Axis Grouping: Tissue); **C.** Gene expression in brain and other tissue using data available GTEx (X-Axis Grouping: Cell type). The size of the black dots represents the total number of cells, while the size of the red dots represents the expression ratio of genes in such cells.

TPM: Transcripts Per Million.

**Reference List**

1 Mishra, A. *et al.* Stroke genetics informs drug discovery and risk prediction across ancestries. *Nature* **611**, 115-+, doi:10.1038/s41586-022-05165-3 (2022).

2 Evangelou, E. *et al.* Genetic analysis of over 1 million people identifies 535 new loci associated with blood pressure traits. *Nature Genetics* **50**, 1412-+, doi:10.1038/s41588-018-0205-x (2018).

3 Nielsen, J. B. *et al.* Biobank-driven genomic discovery yields new insight into atrial fibrillation biology. *Nature Genetics* **50**, 1234-+, doi:10.1038/s41588-018-0171-3 (2018).

4 Xue, A. *et al.* Genome-wide association analyses identify 143 risk variants and putative regulatory mechanisms for type 2 diabetes. *Nature Communications* **9**, doi:10.1038/s41467-018-04951-w (2018).

5 Yengo, L. *et al.* Meta-analysis of genome-wide association studies for height and body mass index in ∼700 000 individuals of European ancestry. *Human Molecular Genetics* **27**, 3641-3649, doi:10.1093/hmg/ddy271 (2018).

6 Suhre, K. *et al.* Connecting genetic risk to disease end points through the human blood plasma proteome. *Nature Communications* **8**, doi:10.1038/ncomms14357 (2017).
